# Comparisons of lanthanide/actinide +2 ions in a tris(aryloxide)arene coordination environment†
†Electronic supplementary information (ESI) available: Additional computational details, spectroscopic information, crystallographic data collection, structure solution, and refinement (PDF), X-ray diffraction details of compounds **1-Ln** (Ln = Nd, Gd, Dy, and Er), **2-Nd**, **2-Ln/3-Ln** (Ln = Gd, Dy, Er), **2-Dy/4-Dy**, and **5-Dy/6-Dy**. CCDC (CIF, 1538987–1538995 and 1566075 for **2-Dy**/**3-Dy**), and DFT-optimized structural coordinates for **2-Nd** and **2-Gd**. For ESI and crystallographic data in CIF or other electronic format see DOI: 10.1039/c7sc02337e



**DOI:** 10.1039/c7sc02337e

**Published:** 2017-09-07

**Authors:** Megan E. Fieser, Chad T. Palumbo, Henry S. La Pierre, Dominik P. Halter, Vamsee K. Voora, Joseph W. Ziller, Filipp Furche, Karsten Meyer, William J. Evans

**Affiliations:** a Department of Chemistry , University of California , Irvine , California 92697-2025 , USA . Email: wevans@uci.edu ; Email: filipp.furche@uci.edu; b Department of Chemistry and Pharmacy , Inorganic Chemistry , Friedrich-Alexander-University Erlangen-Nürnberg (FAU) , Egerlandstrasse 1 , D-91058 Erlangen , Germany . Email: karsten.meyer@fau.de

## Abstract

A new series of Ln^3+^ and Ln^2+^ complexes has been synthesized using the tris(aryloxide)arene ligand system, ((^Ad,Me^ArO)_3_mes)^3–^, recently used to isolate a complex of U^2+^. The triphenol precursor, (^Ad,Me^ArOH)_3_mes, reacts with the Ln^3+^ amides, Ln(NR_2_)_3_ (R = SiMe_3_), to form a series of [((^Ad,Me^ArO)_3_mes)Ln] complexes, **1-Ln**. Crystallographic characterization was achieved for Ln = Nd, Gd, Dy, and Er. The complexes **1-Ln** can be reduced with potassium graphite in the presence of 2.2.2-cryptand (crypt) to form highly absorbing solutions with properties consistent with Ln^2+^ complexes, [K(crypt)][((^Ad,Me^ArO)_3_mes)Ln], **2-Ln**. The synthesis of the Nd^2+^ complex [K(crypt)][((^Ad,Me^ArO)_3_mes)Nd], **2-Nd**, was unambiguously confirmed by X-ray crystallography. In the case of the other lanthanides, crystals were found to contain mixtures of **2-Ln** co-crystallized with either a Ln^3+^ hydride complex, [K(crypt)][((^Ad,Me^ArO)_3_mes)LnH], **3-Ln**, for Ln = Gd, Dy, and Er, or a hydroxide complex, [K(crypt)][((^Ad,Me^ArO)_3_mes)Ln(OH)], **4-Ln**, for Ln = Dy. A Dy^2+^ complex with 18-crown-6 as the potassium chelator, [K(18-crown-6)(THF)_2_][((^Ad,Me^ArO)_3_mes)Dy], **5-Dy**, was isolated as a co-crystallized mixture with the Dy^3+^ hydride complex, [K(18-crown-6)(THF)_2_][((^Ad,Me^ArO)_3_mes)DyH], **6-Dy**. Structural comparisons of **1-Ln** and **2-Ln** are presented with respect to their uranium analogs and correlated with density functional theory calculations on their electronic structures.

## Abstract 2

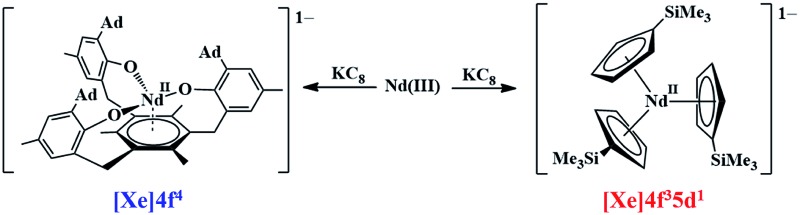
Nd, like U, prefers a f^4^ configuration with the tris(aryloxide)arene ligand rather than the 4f^3^5d^1^ configuration found in tris(cyclopentadienyl) complexes.

## Introduction

Recently, the reduction of the tris(cyclopentadienyl) rare-earth metal complexes, Cp′_3_Ln and Cp′′_3_Ln [Cp′ = C_5_H_4_SiMe_3_; Cp′′ = C_5_H_3_(SiMe_3_)_2_], with KC_8_ in the presence of a chelate such as 2.2.2-cryptand allowed the isolation of the first molecular Ln^2+^ complexes for nine new ions^1–5^ (Ln = La, Ce, Pr, Gd, Tb, Y, Ho, Er, and Lu), eqn (1).^1–10^ These complexes differed from the traditional six 4f^*n*+1^ Ln^2+^ ions (Ln = Eu, Yb, Sm, Tm, Nd, Dy) in that their complexes were much more intensely colored and the metal-(cyclopentadienyl ring centroid) distances in the Ln^2+^ complexes were only *ca.* 0.03 Å longer than the Ln^3+^ analogs. For complexes of traditional Ln^2+^ ions, metal–ligand bond distances are typically 0.12–0.20 Å longer than in +3 analogs. The properties of the new ions were consistent with reduction of the 4f^*n*^ Ln^3+^ precursors to form 4f^*n*^5d^1^ ions rather than the traditional 4f^*n*+1^ ions, a result explained by density functional theory (DFT) calculations.^3–5^
1
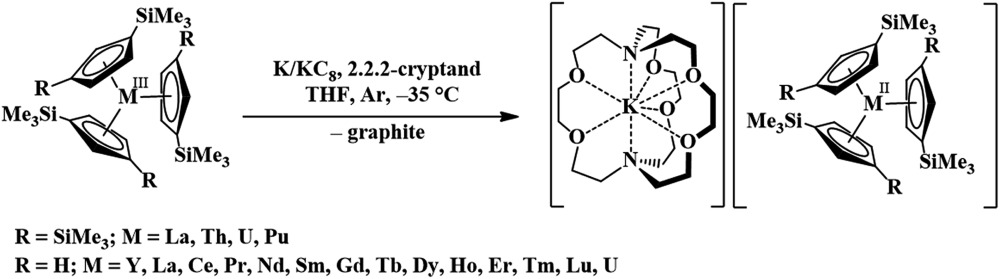



To enable a direct comparison of the new 4f^*n*^5d^1^ ions (La, Ce, Pr, Gd, Tb, Dy, Ho, Er, and Lu) with the traditional 4f^*n*^5d^1^ ions (Eu, Yb, Sm, Tm, Nd, Dy) in a single coordination environment, [K(crypt)][Cp′_3_Ln] complexes were synthesized for the entire lanthanide series (except Pm, which was not studied due to its radioactivity), eqn (1). This revealed that in the (Cp′_3_)^3–^ coordination environment, Nd^2+^ and Dy^2+^ have properties consistent with 4f^*n*^5d^1^ ground states, instead of the 4f^*n*+1^ ground state in previously identified Nd^2+^ and Dy^2+^ complexes. These ions therefore are not traditional 4f^*n*+1^ Ln^2+^ ions, but are configurational crossover ions that can have a variable electronic ground state depending on the ligand environment. This was an unusual result in molecular lanthanide chemistry, given that the limited radial extension of the 4f orbitals generally precludes ligand influences on the electronic configuration. The [K(crypt)][Cp′_3_Ln] results suggest that there are now three classes of Ln^2+^ ions: traditional 4f^*n*+1^ ions, Ln = Eu, Yb, Sm, and Tm, the new 4f^*n*^5d^1^ ions, Ln = La, Ce, Pr, Gd, Tb, Ho, Er, and Lu, and the configurational crossover ions, Ln = Nd and Dy, which can have either 4f^*n+*1^ or 4f^*n*^5d^1^ configurations depending on the coordination environment. Since these groupings arise only from the (Cp′_3_)^3–^ ligand set, it was desirable to find other ligand environments for comparison.

The first crystallographically-characterized U^2+^ complex, [K(crypt)][Cp′_3_U], was also obtained *via*eqn (1).^6^ Analyses of this complex by X-ray crystallography, UV-visible spectroscopy, and DFT were consistent with a quintet 5f^3^6d^1^ ground state for U^2+^ in this coordination environment and the complex displayed properties similar to those of the complexes with 4f^*n*^5d^1^ Ln^2+^ ions. Shortly thereafter, a second U^2+^ complex was reported: the tris(aryloxide)arene U^3+^ complex, [((^Ad,Me^ArO)_3_mes)U], **1-U**, could be reduced to the U^2+^ complex, [K(crypt)][((^Ad,Me^ArO)_3_mes)U], **2-U**, eqn (2).^11,12^
2
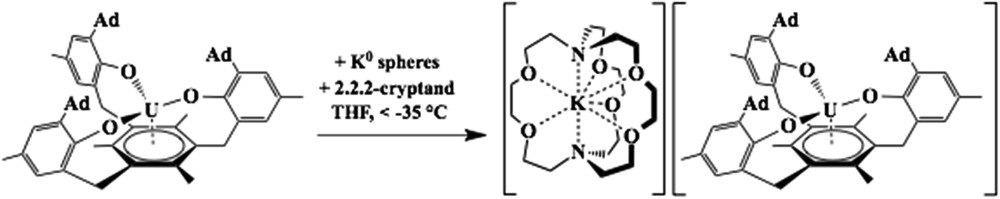



Previous DFT studies on **1-U** revealed two SOMOs with δ backbonding interactions with f orbitals and one SOMO containing a non-bonding uranium 5f electron; **2-U** is similar except there are two non-bonding uranium 5f electrons. Hence, computational analysis of **2-U** was consistent with an *S* = 2, 5f^4^ ground state for U^2+^. Experimental support for the predicted 5f^4^ electronic ground state was obtained by X-band EPR spectroscopy as well as solid-state and solution-phase magnetochemical studies.

The isolation of two U^2+^ complexes with different ground state configurations due to their respective coordination environments indicates that uranium should likewise fit into the configurational crossover class of +2 ions described above for the lanthanides. Since uranium is a congener of neodymium, the suggested classification has some periodic consistency. These results also suggested that in the case of Ln^2+^ ions, a comparative study with both ligand environments, [(^Ad,Me^ArO)_3_mes]^3–^ and [Cp′]^3–^, may shed light on the nature of configurational crossover. To explore this possibility, the synthesis of complexes of new Ln^2+^ ions with the [(^Ad,Me^ArO)_3_mes]^3–^ ligand was pursued. Numerous Ln^3+^ aryloxide complexes have been previously reported in the literature.^13–34^ The synthesis and structural characterization of Ln^3+^ complexes of the [(^Ad,Me^ArO)_3_mes]^3–^ ligand are reported here as well as their reduction chemistry. This has led to highly reactive Ln^2+^ complexes that often co-crystallize either with Ln^3+^ hydride or Ln^3+^ hydroxide byproducts. DFT analysis is used to evaluate the electronic structures and make comparisons with uranium.

## Results and discussion

### Synthesis and structure of the Ln^3+^ complexes [((^Ad,Me^ArO)_3_mes)Ln], **1-Ln**

**Fig. 1:**
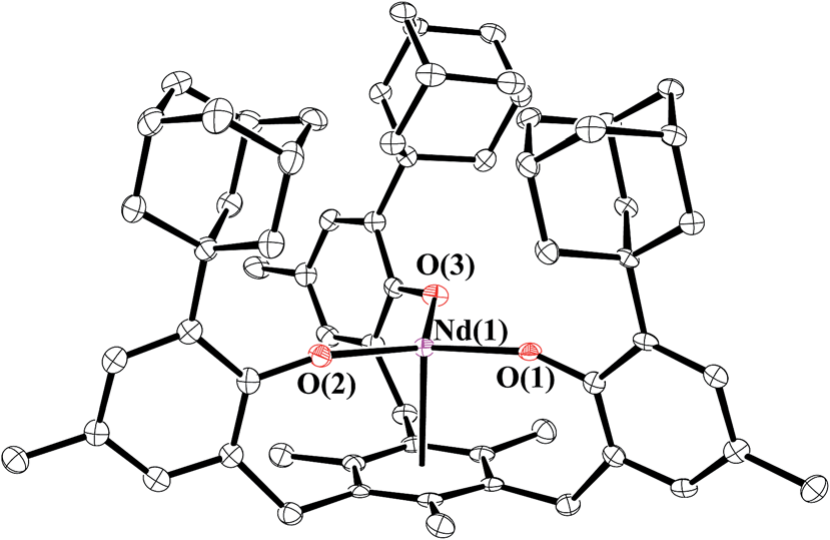
Molecular structure of [((^Ad,Me^ArO)_3_mes)Nd], **1-Nd**, with thermal ellipsoids drawn at the 50% probability level. Hydrogen atoms are omitted for clarity.

The trivalent complexes, [((^Ad,Me^ArO)_3_mes)Ln], **1-Ln** (Ln = Nd, Gd, Dy, and Er), were synthesized by protonolysis of [Ln(N(SiMe_3_)_2_)_3_] with the tris(phenol), (^Ad,Me^ArOH)_3_mes, eqn (3), and identified by X-ray crystallography, Fig. 1. The Gd, Dy, and Er complexes crystallize in the *P*2_1_/*c* space group and are isomorphous. **1-Nd** also crystallizes in *P*2_1_/*c* and is similar in structure, but is not isomorphous with the other **1-Ln** compounds (see ESI† for details). In comparison, **1-U** crystallizes in *P*1.3
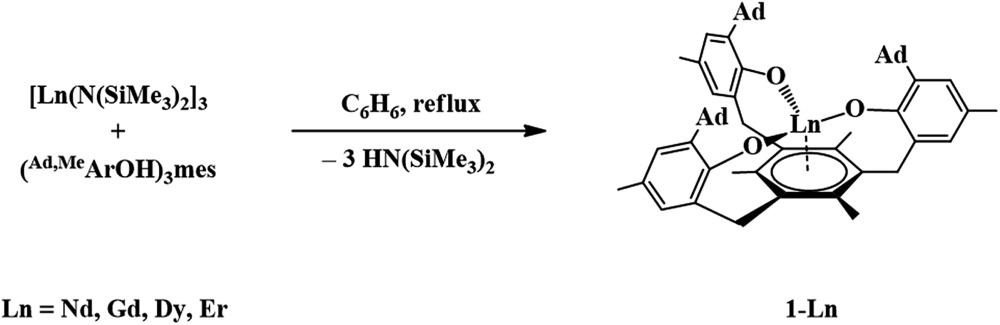



The structural parameters of **1-Ln** follow a regular trend based on the metal ionic radii, Table 1. Hence, the M–O distances and the M–(arene ring centroid) distances decrease regularly from Nd to Er as the size of the Ln^3+^ ion decreases. The Ln–O distances for **1-Ln** fall in the range of reported Ln–O(aryloxide) distances for complexes such as [Ln(OC_6_H_3_^*t*^Bu_2_-2,6)_3_(THF)_3_] (Ln = Nd,^35^ Gd,^36^ Er^37^), [Dy(OC_6_H_3_^*i*^Pr_2_-2,6)_3_(DME)_2_],^38^ as well as other rare earth aryloxide complexes.^13–34^ In contrast, the M–(arene centroid) distances of **1-Ln** are significantly shorter than those reported for Ln^3+^ arene complexes such as (arene)Ln[(*μ*-Cl)_2_AlCl_2_]_3_.^39–50^ For example, (*η* ^6^-1,3,5-C_6_H_3_Me_3_)Nd[(AlCl_4_)_3_]^42^ has a 2.566 Å Ln–(arene centroid) distance compared to 2.489 Å for **1-Nd**.

**Table 1:** Selected bond lengths (Å) and angles (°) of **1-Ln** and **1-U** listed in order of decreasing ionic radius

Metal	Six coordinate ionic radius^*a*^	M–O range	M–O avg	M–C_6_ (ring centroid)	M out of plane^*b*^	C_6_ torsion angle^*c*^
U	1.025	2.158(2)-2.178(2)	2.17(1)	2.35	0.475(2)	6.8
Nd	0.983	2.172(3)-2.200(2)	2.19(1)	2.489	0.268	5.6
Gd	0.938	2.132(2)-2.134(2)	2.133(1)	2.413	0.416	8.3
Dy	0.912	2.093(3)-2.095(3)	2.094(1)	2.368	0.443	8.1
Er	0.89	2.078(2)-2.081(2)	2.079(1)	2.336	0.477	7.9

^*a*^From Shannon.^51^

^*b*^Distance of M from the plane defined by the three O atoms of the ((^Ad,Me^ArO)_3_mes)^3–^ ligand.

^*c*^The largest dihedral angle between adjacent three-carbon planes in the mesitylene ring.

Table 1 also shows that the distances for **1-U** do not match those of **1-Ln** in terms of radial size and metal–ligand distance. Both the U–O and U**–**(arene centroid) distances of the U^3+^ complex are shorter than those of the lanthanides. This difference can be rationalized by greater orbital overlap between the ligand orbitals and the 5f *vs.* the 4f metal orbitals. Regardless of these differences, the average C–C bond distances in the arene ring are within error of those of the free ligand, (^Ad,Me^ArOH)_3_mes, whose structure was determined as part of this study (see ESI†). Thus there is no evidence of reduction of the arene ring.

### Reduction reactions

**Fig. 2:**
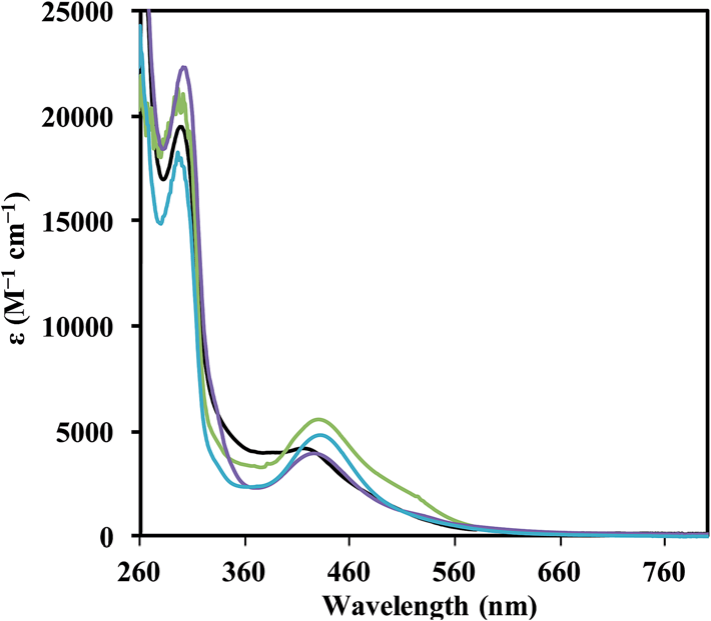
UV-visible spectra of [K(chelate)][((^Ad,Me^ArO)_3_mes)Ln] with Ln = Nd (black), Gd (purple), Er (green), and Dy (blue), recorded in THF at 298 K. The solutions were generated from crystals of **2-Nd**, **2-Ln/3-Ln** (Ln = Gd, Er), and **5-Dy/6-Dy**. Extinction coefficients, *ε*, for **2-Ln** (Nd, Gd, Er) and **5-Dy** were calculated using concentrations of Ln^2+^ estimated using Ln(1) occupancy from the crystallographic data.

Reduction of each **1-Ln** complex was carried out in 1 : 1 THF/C_6_H_6_ with potassium graphite (KC_8_) in the presence of 2.2.2-cryptand (crypt). In each case, highly absorbing red-colored solutions were obtained that were reminiscent of the intensely-colored solutions produced in the reductions of the Cp′_3_Ln complexes in eqn (1). The UV-visible electronic absorption spectra of these dark solutions, as shown in Fig. 2, differ greatly from the line-like spectra typical of Ln^3+^ complexes (see **1-Nd**, Fig. S11†). Each complex has a strong broad absorption band in the visible region with the following maxima (*λ*_max_, *ε*): Nd (416 nm, 4200 M^–1^ cm^–1^), Gd (426 nm, 4000 M^–1^ cm^–1^), Dy (431 nm, 4900 M^–1^ cm^–1^), and Er (430 nm, 5600 M^–1^ cm^–1^). The absorption energies and extinction coefficients of **2-Ln** are similar to those reported for the [K(crypt)][Cp′_3_Ln] complexes (*λ*_max_, *ε*): Nd (420 nm, 4700 M^–1^ cm^–1^), Gd (430 nm, 4400 M^–1^ cm^–1^), Dy (483 nm, 3400 M^–1^ cm^–1^), and Er (502 nm, 4000 M^–1^ cm^–1^). Although all of these absorption bands for **2-Ln** are broad, they appear to follow a trend in which the absorption energy decreases with increasing atomic number. Single crystals of the reduction products were obtained for Ln = Nd, Gd, Er, and Dy and are described below.

### Neodymium

**Fig. 3:**
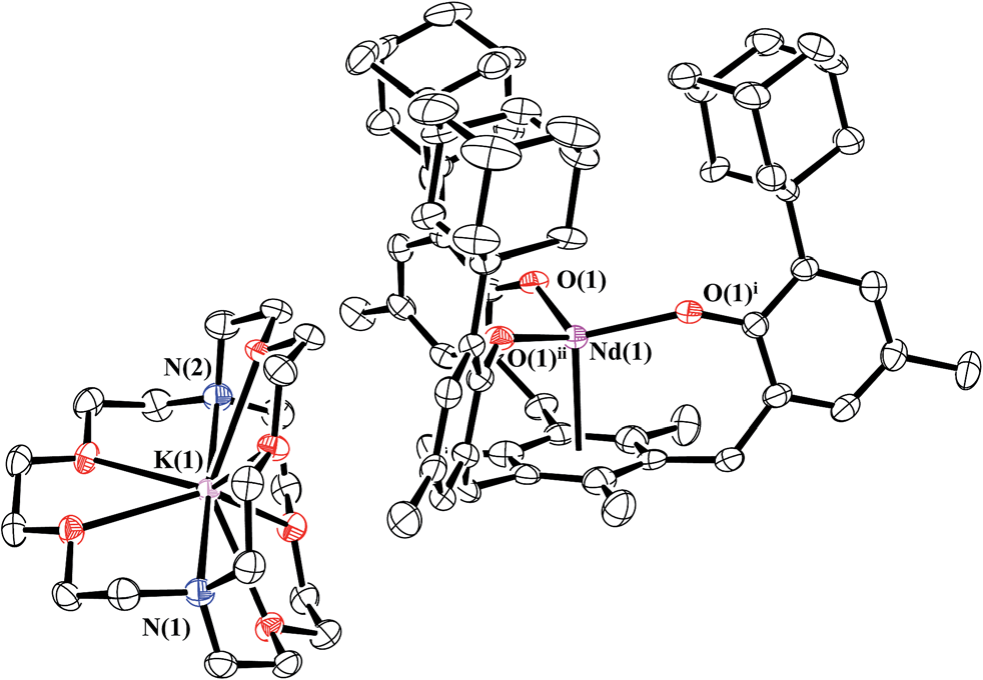
Molecular structure of [K(crypt)][((^Ad,Me^ArO)_3_mes)Nd], **2-Nd**, drawn at the 50% probability level. Hydrogen atoms are omitted for clarity.

Reduction of **1-Nd** produced a new example of a Nd^2+^ complex, [K(crypt)][((^Ad,Me^ArO)_3_mes)Nd], **2-Nd**, eqn (4), which was confirmed by single-crystal X-ray diffraction, Fig. 3. Crystals of **2-Nd** form in space group *P*2_1_/*c* and are isomorphous with crystals of the U^2+^ complex, [K(crypt)][((^Ad,Me^ArO)_3_mes)U], **2-U** (see ESI†).4
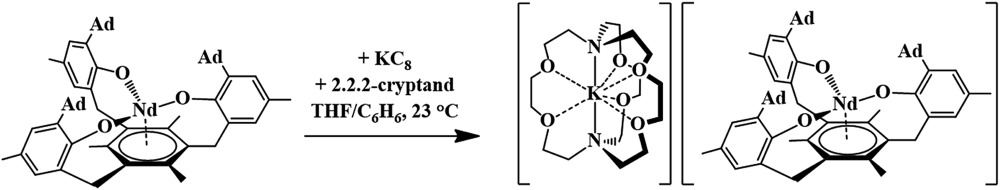



### Gadolinium and erbium

**Fig. 4:**
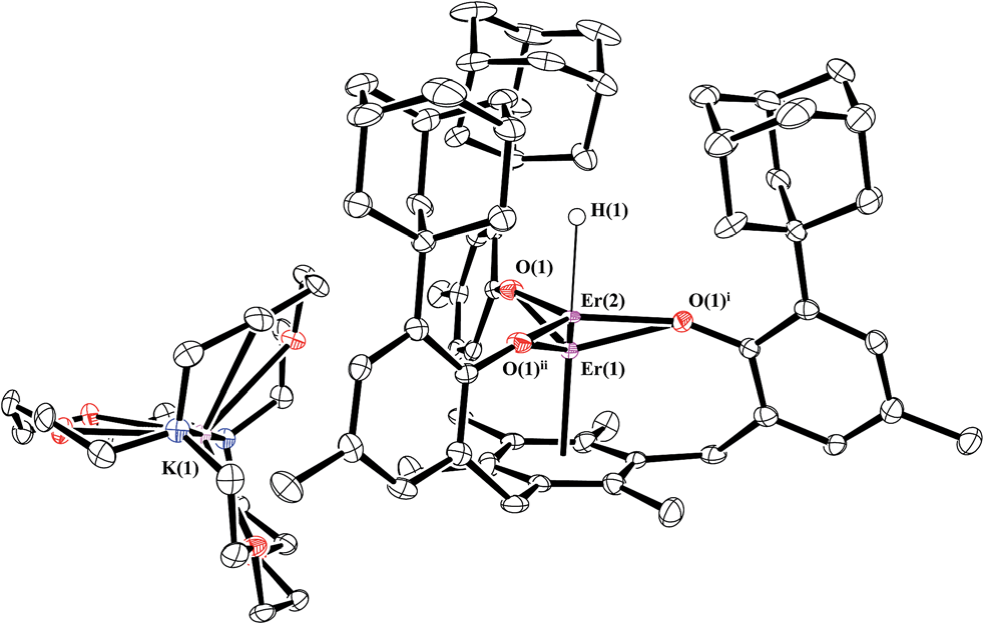
Molecular structure of [K(crypt)][((^Ad,Me^ArO)_3_mes)Er], **2-Er**, and [K(crypt)][((^Ad,Me^ArO)_3_mes)ErH], **3-Er**, which co-crystallize in an approximate 55 : 45 ratio. Thermal ellipsoids are drawn at the 50% probability level and hydrogen atoms, except H(1), are omitted for clarity. Er(1) is the metal position in **2-Er** and Er(2) is the metal position in **3-Er**.

Reductions of **1-Gd** and **1-Er**, performed in a manner analogous to that of eqn (4), produced dark red single crystals suitable for X-ray diffraction that appeared to be isomorphous with **2-Nd** (see ESI†). However, the crystallographic data were best modeled by a mixture of two complexes: the divalent [K(crypt)][((^Ad,Me^ArO)_3_mes)Ln], **2-Ln**, and the trivalent hydride, [K(crypt)][((^Ad,Me^ArO)_3_mes)LnH], **3-Ln**, in a 65 : 35 ratio for Gd and a 55 : 45 ratio for Er, eqn (5), Fig. 4. The metal centers in both **2-Ln** and **3-Ln** lie on a three-fold axis with the same ligand environment, in which Ln(1) represents the metal center for **2-Ln** and Ln(2) represents the metal center for **3-Ln**. Consistent with the presence of a hydride ligand, the reaction of **2-Er/3-Er** with CCl_4_ produced chloroform.^52^
5
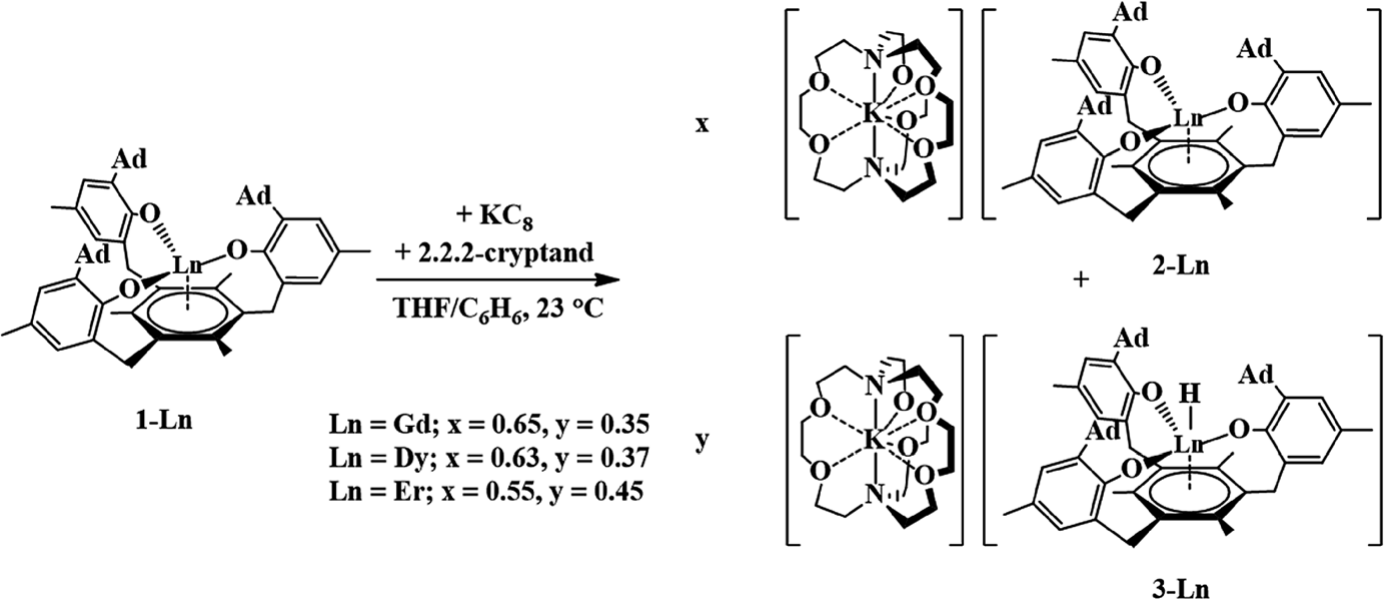



**Fig. 5:**
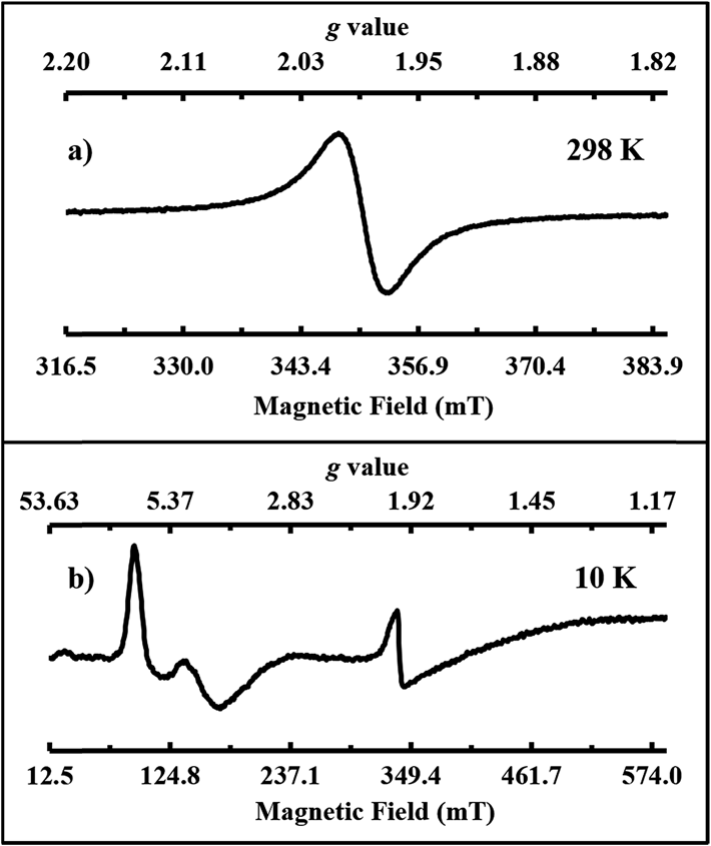
Experimental X-band EPR spectra of single crystals of **2-Gd/3-Gd** dissolved in THF (1 mM) at (a) 298 K (mode: perpendicular; *g*_iso_ = 1.990; *ν* = 9.762 GHz; *P* = 0.0203 mW; modulation amplitude = 0.902 mT) and (b) 10 K (mode: parallel; *g*_1_ = 7.349, *g*_2_ = 4.786, *g*_3_ = 1.977; *ν* = 9.383 GHz; *P* = 2.026 mW; modulation amplitude = 1.002 mT).

Single crystals of **2-Gd/3-Gd** dissolved in THF display a single isotropic signal at *g*_iso_ = 1.990 in the room temperature X-band EPR spectrum, Fig. 5a. This is similar to the X-band EPR spectra of the crystallographically-characterized Gd^2+^ complexes,^4,53^ [K(crypt)][Cp′_3_Gd] and [K(crypt)][Cp′′_2_CpGd] (Cp = C_5_H_5_), which also show isotropic signals at *g*_iso_ = 1.99. A 4f^7^5d^1^ electron configuration has been proposed for those cyclopentadienyl complexes. Thus, the EPR spectrum of **2-Gd** is consistent with a 4f^7^5d^1^ electron configuration for Gd^2+^ in the ((^Ad,Me^ArO)_3_mes)^3–^ ligand coordination, since it is unlikely that an EPR spectrum of a 4f^8^ Gd^2+^ complex would be observable under these conditions. Since a 4f^7^/4f^8^ reduction eliminates a half-filled shell, whereas an 4f^7^ to a 4f^7^5d^1^ reduction does not, the latter process would be favored in this regard. Elimination of the half-filled shell is why the calculated redox potential for a 4f^7^/4f^8^ process is so high, –3.9 V *vs.* SHE,^54^ whereas the observed gadolinium reduction must occur at potentials less negative than –2.9 V *vs.* SHE. The X-band EPR spectrum recorded in frozen THF solution at 10 K, shown in Fig. 5b, is further consistent with the presence of a 4f^7^5d^1^ Gd^2+^ ion. Both the Gd^3+^ and Gd^2+^ species of the co-crystallized sample of **2-Gd** and **3-Gd** can be observed by EPR spectroscopy according to our simulations (see ESI†). The almost axial spectrum of **2-Gd** was simulated with *g* values at *g*_1_ = 7.02, *g*_2_ = 6.85, and *g*_3_ = 3.97.

Although co-crystallization of Ln^3+^ hydrides with the Ln^2+^ complexes complicates the structural analysis (see below), it does suggest that the ((^Ad,Me^ArO)_3_mes)^3–^ ligand set can enhance the bond activation reactivity of these Ln^2+^ ions. C–H bond activation previously has been observed with the Nd^2+^ complex, [(C_5_H_2_^*t*^Bu_3_)_2_Nd(*μ*-I)K(18-crown-6)], which forms [(C_5_H_2_^*t*^Bu_3_)(C_5_H_2_^*t*^Bu_2_CMe_2_CH_2_-*η* ^5^:*κ* ^1^)Nd(*μ*-I)K(18-crown-6)].^55^ This was also found in attempts to form indenyl Ln^2+^ complexes, which led to the indenyl dianion, (C_9_H_6_)^2–^, in [K(crypt)]_2_[(C_9_H_7_)_2_Dy(*μ-η* ^5^:*η* ^1^-C_9_H_6_)]_2_.^53^

### Dysprosium

**Fig. 6:**
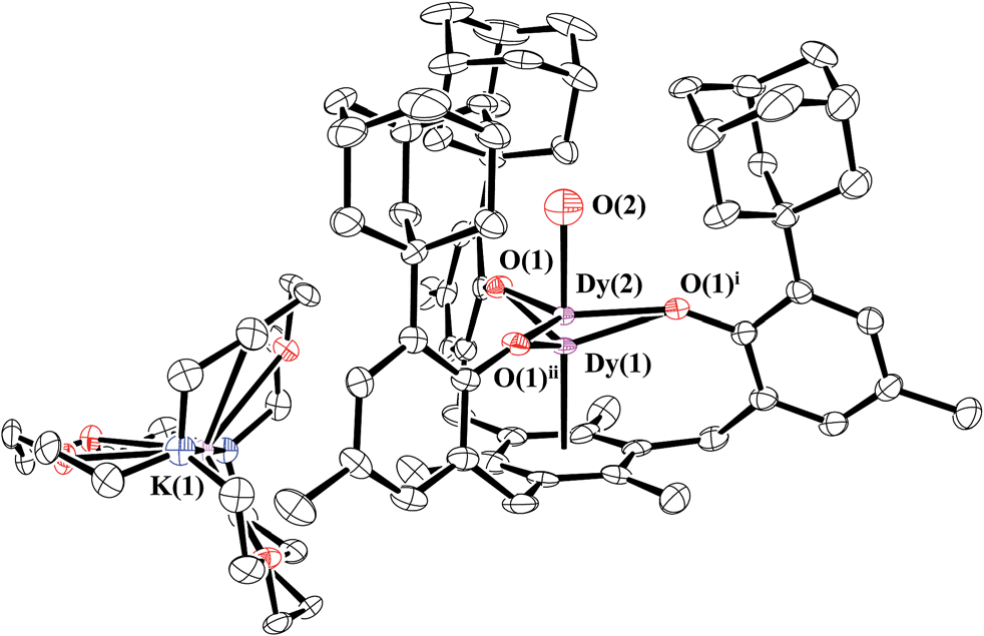
Molecular structure of [K(crypt)][((^Ad,Me^ArO)_3_mes)Dy]/[K(crypt)] [((^Ad,Me^ArO)_3_mes)Dy(OH)], **2-Dy/4-Dy**, with thermal ellipsoids drawn at the 50% probability level. Hydrogen atoms and a disordered ether molecule are omitted for clarity. Dy(1) is the metal position in **2-Dy** and Dy(2) is the metal position in **4-Dy**.

Reduction of **1-Dy** produced dark red crystals suitable for X-ray diffraction that also appeared to be isomorphous with **2-Nd**. Instead, the crystallographic data were best modeled as a mixture of the divalent [K(crypt)][((^Ad,Me^ArO)_3_mes)Dy], **2-Dy**, and the trivalent hydroxide [K(crypt)][((^Ad,Me^ArO)_3_mes)Dy(OH)], **4-Dy**, in a 2 : 3 ratio. Like the **2-Ln/3-Ln** mixtures, **2-Dy** and **4-Dy** lie on a threefold axis with the same ligand environment, Fig. 6, in which Dy(1) represents the metal center for **2-Dy** and Dy(2) represents the metal center for **4-Dy**. The origin of the hydroxide ligand in **4-Dy** is unknown. We include the data on this mixed crystal here, because it does contain a Dy^2+^ complex and shows that the Ln^2+^ complexes can co-crystallize with hydroxides as well as hydrides.

Given the unusual hydroxide result above, the Dy reaction was examined further with 18-crown-6. Reduction of **1-Dy** with K in the presence of 18-crown-6 instead of 2.2.2-cryptand gave a dark colored solution similar to that observed to form the **2-Dy/4-Dy** mixture. Crystallization of this product gave single crystals that were modeled as a 1 : 1 mixture of [K(18-crown-6)(THF)_2_][((^Ad,Me^ArO)_3_mes)Dy], **5-Dy**, and the trivalent hydride, [K(18-crown-6)(THF)_2_)][((^Ad,Me^ArO)_3_mes)DyH], **6-Dy** (see ESI†). This **5-Dy/6-Dy** mixture is analogous to the **2-Ln/3-Ln** mixtures, except that the countercation is [K((18-crown-6)(THF)_2_]^+^ rather than [K(crypt)]^+^.

Subsequently, the reduction of **1-Dy** was re-examined and single crystals containing a mixture of the Dy^2+^ complex and the Dy^3+^ hydride were obtained, *i.e.* **2-Dy/3-Dy**. In this case the ratio of Dy^2+^ to Dy^3+^ hydride was modeled by a 63 : 37 mixture.

### Structural comparisons

Structural data on **2-Nd** and the co-crystallized **2-Gd/3-Gd**, **2-Dy/3-Dy**, **2-Er/3-Er**, **2-Dy/4-Dy**, and **5-Dy/6-Dy** mixtures are given in Table 2, along with the data for **2-U**. In contrast to the data on the Ln^3+^ **1-Ln** complexes shown in Table 1, the structural data on the mixtures presented in Table 2 do not follow the regular changes in distances with radial size for either the Ln^2+^ complexes, **2-Ln** and **5-Dy**, or for the Ln^3+^ complexes, **3-Ln**, **4-Dy**, and **6-Dy**. The substantial differences in the metrical parameters of the [K(crypt)]^+^ and [K(18-crown-6)(THF)_2_]^+^ salts of the [((^Ad,Me^ArO)_3_mes)Dy]^–^ anion, **2-Dy** and **5-Dy**, illustrate the complicated nature of these structural data. As a result, only the metrical data for **2-U** and **2-Nd** will be compared.

**Table 2:** Selected bond lengths (Å) and angles (°) of **2-U**, **2-Nd**, **2-Gd/3-Gd**, **2-Er/3-Er**, **2-Dy/4-Dy**, and **5-Dy/6-Dy**

Metal	M–O	M–Cent	M–C(arene)	M–C(arene) avg	M out of plane^*a*^	O–M–O	Largest C6 torsion angle (°)^*b*^
**2-U**	2.236(4)	2.18	2.597(5), 2.633(5)	2.615	0.668(2)	111.49(8)	5.9
**2-Nd**	2.237(4)	2.366	2.742(6), 2.788(7)	2.765	0.530	114.59(8)	6.2
**2-Gd/3-Gd**	2.203(3)/2.126(3)	2.286/2.863	2.672(5)/3.175, 2.710(6)/3.216	2.691/3.196	0.578/0.001	113.37(8)/120.003(1)	8.8
**2-Dy/3-Dy**	2.182(3)/2.095(3)	2.238/2.837	2.632(4)/3.152, 2.670(5)/3.194	2.651/3.173	0.608/0.009	112.55(8)/119.997(1)	6.8
**2-Dy/4-Dy**	2.222(3)/2.125(3)	2.177/2.683	2.586(5)/3.101, 2.605(6)/3.127	2.596/3.113	0.652/0.055	111.77(8)/119.93	6.3
**5-Dy/6-Dy**	2.16(3)/2.09(2)	2.232/2.789	2.592(4)/3.058, 2.603(4)/3.068, 2.613(4)/3.096, 2.641(4)/3.128, 2.665(4)/3.169, 2.728(4)/3.234	2.64(5)/3.13(6)	0.556/0.001	114.39(12)/118.61, 115.22(13)/121.78, 111.28(12)/119.61	8.7
**2-Er/3-Er**	2.172(3)/2.077(3)	2.200/2.854	2.602(4)/3.170, 2.634(4)/3.205	2.618/3.188	0.637/0.017	111.77(7)/119.993(2)	5.7

^*a*^Distance of M from the plane defined by the three O atoms of the ((^Ad,Me^ArO)_3_mes)^3–^ ligand.

^*b*^The largest dihedral angle between adjacent three-carbon planes in the mesitylene ring.

A comparison of the divalent complexes **2-Nd** and **2-U**, along with their trivalent analogs, is given in Table 3. The structural data on **2-Nd** show that the metal center is 0.123 Å closer to the arene centroid than in **1-Nd**. This change is not as large as the 0.17 Å difference between **1-U** and **2-U**, which is likely due to the limited radial extension of the 4f orbitals *vs.* the 5f orbitals.^56^ Just as in **2-U**, the arene carbon atoms are approximately planar in **2-Nd** and the C–C(arene) bond lengths only increase by approximately 0.01 Å. This is consistent with reduction of the metal and not the arene. As analyzed for uranium, the contraction of the M–(arene centroid) distance between **1-Nd** and **2-Nd** could suggest a greater interaction between the metal and arene due to a change in charge distribution.

**Table 3:** Differences (*Δ*) in bond distances (Å) and angles (°) between **2-Nd** and **2-U** and their trivalent analogs, **1-Nd** and **1-U**, respectively

Metal	*Δ*(M–O)	*Δ*(M–Cent)	*Δ*(M out of plane)^*a*^	*Δ*(largest C6 torsion angle)
**U**	0.068	–0.170	0.193	–0.9
**Nd**	0.050	–0.123	0.262	0.6

^*a*^Distance of M from the plane defined by the three O atoms of the ((^Ad,Me^ArO)_3_mes)^3–^ ligand.

### Theoretical insight

**Fig. 7:**
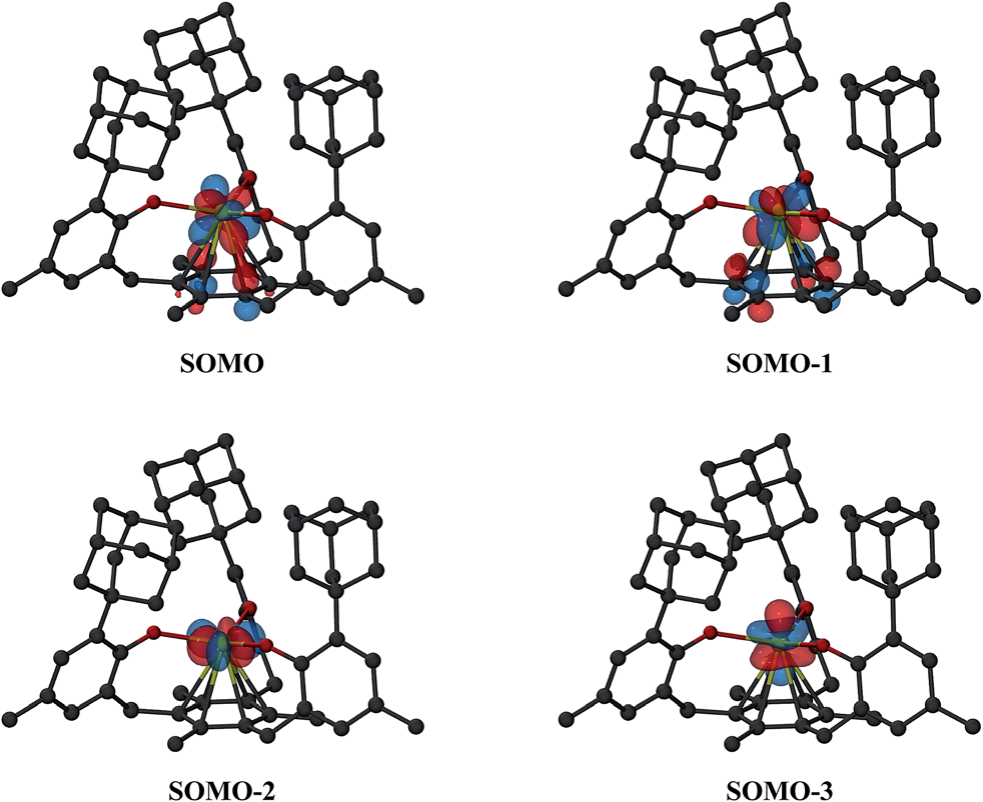
Isosurfaces for the four highest singly-occupied molecular orbitals of **2-Nd** corresponding to a contour value of 0.05. Hydrogen atoms are omitted for clarity.

Density functional theory (DFT) calculations using the Tao–Perdew–Staroverov–Scuseria (TPSS) functional^57^ and mixed basis sets were carried out on **1-Nd**, **2-Nd**, and **2-Gd** (see ESI for further details†).^58,59^ For **1-Nd** and **2-Nd**, the calculated structural parameters match those observed within 0.04 Å (Table S5, ESI†). The three valence electrons of **1-Nd** occupy predominantly 4f-type orbitals with little observable interaction with the mesitylene ring. This differs from **1-U** as expected for a 4f *vs.* 5f system.^11^ Calculations on **2-Nd** suggest a quintet ground state with two electrons in f orbitals and two electrons in f/π* orbitals of *δ* symmetry (see Tables S6 and S7†); the corresponding four SOMOs are shown in Fig. 7. This orbital picture resembles that of **2-U**.^12^ The lowest unoccupied orbital with d-orbital character for **2-Nd** is about 2.9 eV above the HOMO and has d_z_^2^ character (see Fig. S11, ESI†). Hence, the DFT calculations suggest that the ((^Ad,Me^ArO)_3_mes)^3–^ ligand system favors a formal 4f^4^ electron configuration for Nd^2+^ rather than a 4f^3^5d^1^ configuration postulated for Nd^2+^ in the (Cp′_3_)^3–^ environment.^5^ This assignment is consistent with Nd^2+^ being a configurational crossover ion and is further supported by the 5f^4^ configuration found for [K(crypt)][((^Ad,Me^ArO)_3_mes)U], **2-U**, *vs.* the 5f^3^6d^1^ configurations for [K(crypt)][Cp′_3_U]^6^ and [K(crypt)][Cp′′_3_U].^7^.

**Fig. 8:**
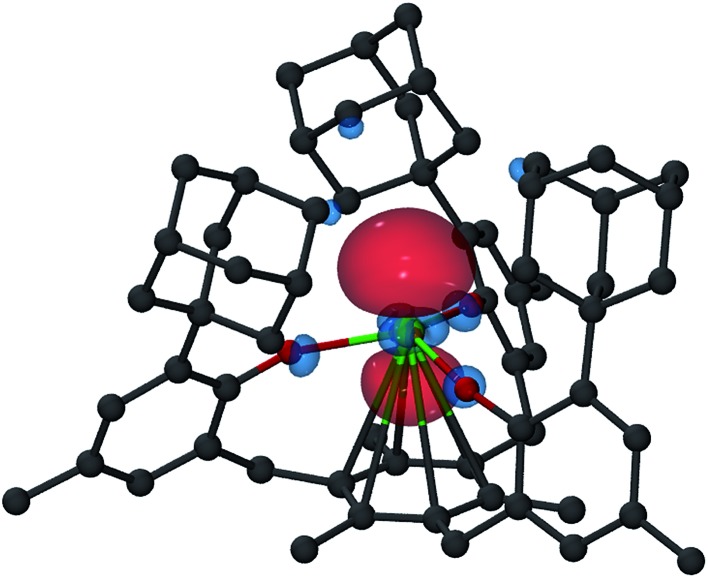
Isosurface of the highest SOMO of nonet **2-Gd** with a contour value of 0.05. Hydrogen atoms are omitted for clarity.

DFT calculations on **2-Gd** proved to be more challenging. The ground state of **2-Gd** is a nonet (8 unpaired electrons) with a 4f^7^(5d/6s)^1^ configuration for the Gd atom (Fig. 8). This result is similar to the 4f^7^5d^1^ configuration observed for [K(crypt)][Cp′_3_Gd], except that the SOMO has 6s as well as 5d character. A nonet ground state is also supported by the observable EPR spectrum for **2-Gd** (Fig. 5). However, the computed metal-arene bond-distance (3.17 Å) is larger than the experimentally observed bond-distance (2.29 Å) and the calculated metal out-of-plane distortion (–0.28 Å) is in a direction opposite to the experimental value (0.578 Å), see Table S5,† indicating that the DFT results for **2-Gd** need to be interpreted with caution. The potential energy profile along the Gd out-of-plane distortion is fairly shallow and has several minima with different electronic character, and the DFT picture may not adequately capture the multi-configurational nature of the nonet ground state. In any case, it appears that the ((^Ad,Me^ArO)_3_mes)^3–^ ligand system can favor 4f^*n*+1^ over 4f^*n*^5d^1^ with the configurational crossover ion, Nd^2+^, but this effect is not strong enough to overcome the stabilization derived from a 4f^7^ half-filled shell in Gd^2+^.

## Conclusion

Tris(aryloxide) arene lanthanide(iii) complexes, [((^Ad,Me^ArO)_3_mes)Ln], **1-Ln**, analogous to [((^Ad,Me^ArO)_3_mes)U], **1-U**,^11^ have been synthesized and characterized by single-crystal X-ray diffraction for Ln = Nd, Gd, Dy, and Er. The four trivalent Ln complexes show structural regularity in metal ligand distances based on their decreasing radial size from Nd to Er. Complex **1-U** appears to have greater interaction with the tris(aryloxide)arene ligand consistent with greater radial extension of the 5f orbitals. Reduction of **1-Ln** generates four new Ln^2+^ complexes, [K(crypt)][((^Ad,Me^ArO)_3_mes)Ln], **2-Ln**, for Nd, Gd, Dy, and Er as well as the 18-crown-6 variant, [K(18-crown-6)(THF)_2_][((^Ad,Me^ArO)_3_mes)Dy], **5-Dy**. **2-Gd**, **2-Er**, and **5-Dy** co-crystallize with Ln^3+^ hydrides, [K(crypt)][((^Ad,Me^ArO)_3_mes)LnH], **3-Ln**, or [K((18-crown-6)(THF)_2_)][((^Ad,Me^ArO)_3_mes)DyH], **6-Dy**. This suggests that the ((^Ad,Me^ArO)_3_mes)^3–^ ligand environment is especially effective at promoting high reactivity.

DFT calculations indicate that the one Ln^2+^ complex isolated without Ln^3+^ co-crystallization, **2-Nd**, appears to have a 4f^4^ electron configuration with two electrons in 4f/π* orbitals and two electrons in other 4f orbitals. This contrasts with the 4f^3^5d^1^ configuration of [Cp′_3_Nd]^1–^ and is consistent with Nd^2+^ being a configurational crossover ion. Comparison of **2-Nd** with congeneric and isomorphous **2-U** shows closer interaction of the metal with the ligand in the case of the 5f *vs.* 4f metal, which is consistent with the relative radial extensions of these orbitals. EPR data and DFT calculations on [K(crypt)][((^Ad,Me^ArO)_3_mes)Gd]/[K(crypt)][((^Ad,Me^ArO)_3_mes)GdH], **2-Gd**/**3-Gd**, tentatively suggest a 4f^7^5d^1^ electron configuration that retains a half-filled 4f shell for Gd^2+^ in the [(^Ad,Me^ArO)_3_mes]^3–^ coordination environment, although the poor agreement of the DFT metal-arene bond distance with the X-ray data merits further investigation. Overall, the results suggest that the [(^Ad,Me^ArO)_3_mes]^3–^ ligand has considerable flexibility in binding heavy metals.

### Experimental details

The syntheses and manipulations described below were conducted under an argon atmosphere with rigorous exclusion of air and water using glovebox, vacuum line, and Schlenk techniques. Solvents were sparged with ultrahigh purity (UHP) grade argon (Airgas) and passed through columns containing Q-5 and molecular sieves before use. NMR solvents (Cambridge Isotope Laboratories) were dried over NaK/benzophenone, degassed by three freeze–pump–thaw cycles, and vacuum-transferred before use. [Ln(N(SiMe_3_)_2_)_3_] (Ln = Nd, Gd, Dy, Er),^60^ KC_8_,^61^ and (^Ad,Me^ArOH)_3_mes,^11^ were prepared according to literature. 2.2.2-Cryptand, 4,7,13,16,21,24-hexaoxa-1,10-diazabicyclo[8.8.8]hexacosane (Acros Organics), was placed under vacuum (10^–3^ Torr) for 12 h before use. 18-Crown-6 (Aldrich) was sublimed before use. ^1^H NMR (500 MHz) spectra were obtained on a Bruker GN500 or CRYO500 MHz spectrometer at 298 K. IR samples were prepared as KBr pellets and the spectra were obtained on either a Varian 1000 or Jasco 4700 FT-IR spectrometer. Elemental analyses were performed on a PerkinElmer 2400 series II CHNS elemental analyzer. Electronic absorption spectra were obtained in THF or benzene at 298 K using a Varian Cary 50 Scan UV-vis or Jasco V-670 UV/Vis/NIR/MIR absorption spectrometer. EPR spectra were collected using X-band frequency (9.3–9.8 GHz) on a Bruker EMX spectrometer equipped with an ER041XG microwave bridge and the magnetic field was calibrated with DPPH (*g* = 2.0036).

### [((^Ad,Me^ArO)_3_mes)Nd], **1-Nd**

In an argon-filled glovebox, a sealable 100 mL side-arm Schlenk flask equipped with a greaseless stopcock was charged with a solution of (^Ad,Me^ArOH)_3_mes (256 mg, 0.290 mmol) in benzene (40 mL) and a magnetic stir bar. A solution of [Nd(N(SiMe_3_)_2_)_3_] (251 mg, 0.305 mmol) in benzene (40 mL) was slowly added to the stirred solution. Higher concentrations resulted in gel-like precipitates and low yields. The flask was attached to a Schlenk line and the mixture was stirred and heated at reflux for 18 h. The flask was brought back into the glovebox, the solution was filtered, and the solvent was removed from the colorless filtrate under vacuum. The resulting pale-blue solid was washed with hexanes then extracted into benzene (15 mL) and filtered. Toluene (5 mL) was added to the filtrate and removal of solvent under vacuum afforded **1-Nd** as a pale-blue powder (163 mg, 56%). Blue single crystals of **1-Nd**, suitable for X-ray diffraction, were grown from slow evaporation of a C_6_D_6_ solution. ^1^H NMR (C_6_D_6_): *δ* 16.0 (s, 3H), 10.7 (s, 3H), 7.0 (s, 9H), 3.5 (br s, 18H), 1.1 (br s, 9H), 1.00 (br s, 6H), –6.2 (s, 9H), –7.2 (s, 9H), –16.3 (s, 9H). IR: 3074w, 2898s, 2845w, 2675w, 2652w, 1730w, 1601w, 1568w, 1492m, 1445s, 1380m, 1340m, 1305m, 1285s, 1245s, 1205m, 1184m, 1160m, 1113w, 1100m, 1066s, 1019m, 980m, 960m, 915m, 886m, 835s, 820s, 808s, 737m, 733s, 729m, 694m, 679w, 631w. Anal. calcd for C_63_H_75_NdO_3_: C, 73.86; H, 7.38. Found: C, 74.09; H, 7.35.

### [((^Ad,Me^ArO)_3_mes)Gd], **1-Gd**

As described for **1-Nd**, a solution of [Gd(N(SiMe_3_)_2_)_3_] (73 mg, 0.115 mmol) in benzene (40 mL) was slowly added to a stirred solution of (^Ad,Me^ArOH)_3_mes (100 mg, 0.113 mmol) in benzene (30 mL) to afford **1-Gd** as an off-white solid (104 mg, 86%). Colorless single crystals of **1-Gd**, suitable for X-ray diffraction, were grown from an Et_2_O/hexane solution at –35 °C. IR: 3067w, 3017w, 2960s, 2897s, 2849s, 2732w, 2672w, 2652w, 1739w, 1605w, 1568w, 1545w, 1494m 1453s, 1377m, 1366m, 1354m, 1341m, 1317s, 1308s, 1284s, 1252s, 1209s, 1184m, 1161m, 1116w, 1102m, 1068m, 1037w, 1017m, 983m, 960m, 937w, 911m, 915m, 888m, 881m, 858s, 835s, 820s, 809s, 765m, 748m, 729m, 694m, 683w, 668w, 653w, 646w, 643w, 607w. Anal. calcd for C_63_H_75_GdO_3_: C, 72.93; H, 7.29. Found: C, 73.04; H, 7.26.

### [((^Ad,Me^ArO)_3_mes)Dy], **1-Dy**

As described for **1-Nd**, a solution of [Dy(N(SiMe_3_)_2_)_3_] (298 mg, 0.354 mmol) in benzene (20 mL) was slowly added to a stirred solution of (^Ad,Me^ArOH)_3_mes (303 mg, 0.343 mmol) in benzene (30 mL) to afford **1-Dy** as an off-white solid (236 mg, 66%). Colorless single crystals of **1-Dy**, suitable for X-ray diffraction, were grown from an Et_2_O/hexane solution at –35 °C. IR: 3068w, 2946s, 2899s, 2844s, 2725w, 2675w, 2653w, 1745w, 1605w, 1568w, 1545w, 1495w, 1447s, 1379m, 1366m, 1354m, 1341m, 1315m, 1306m, 1287s, 1250s, 1208m, 1186m, 1161m, 1114w, 1101m, 1068m, 1035m, 1020m, 980m, 963m, 937w, 923m, 917m, 878w, 880w, 845m, 835s, 822s, 809s, 767m, 748m, 728m, 693w, 674s, 666w, 650w, 631w, 606w. Anal. calcd for C_63_H_75_DyO_3_: C, 72.56; H, 7.25. Found: C, 72.28; H, 7.31.

### [((^Ad,Me^ArO)_3_mes)Er], **1-Er**

As described for **1-Nd**, a solution of [Er(N(SiMe_3_)_2_)_3_] (78 mg, 0.120 mmol) in benzene (20 mL) was slowly added to a stirred solution of (^Ad,Me^ArOH)_3_mes (100 mg, 0.113 mmol) in benzene (20 mL) to afford **1-Er** as a pink solid (70 mg, 59%). Pale pink single crystals of **1-Er**, suitable for X-ray diffraction, were grown from an Et2O/hexane solution at –35 °C. IR: 3075w, 2898s, 2845s, 2675w, 2653w, 1733w, 1601w, 1568w, 1542w, 1492m, 1447s, 1381m, 1341m, 1305m, 1286s, 1246s, 1207m, 1185m, 1161m, 1117w, 1100m, 1066s, 1019m, 980w, 961w, 915m, 878w, 856m, 836s, 821s, 809s, 766m, 748m, 735m, 695m, 680w, 652w, 631w. Anal. calcd for C_63_H_75_ErO_3_: C, 72.23; H, 7.22. Found: C, 72.88; H, 7.80.

### [K(crypt)][((^Ad,Me^ArO)_3_mes)Nd], **2-Nd**

In an argon-filled glovebox, [((^Ad,Me^ArO)_3_mes)Nd], **1-Nd** (60 mg, 0.059 mmol), was combined with 2.2.2-cryptand (22 mg, 0.058 mmol) in a vial containing a magnetic stir bar and dissolved in 1 : 1 THF/C_6_H_6_ (4 mL). KC_8_ (15 mg, 0.11 mmol) was quickly added to the pale blue solution. The reaction immediately turned brown. After 2 min, the solution was filtered to remove the graphite. The resulting red-orange solution was layered with Et_2_O (15 mL) and stored at –35 °C for 48 h to produce brown/orange crystals of **2-Nd** suitable for X-ray diffraction (16 mg, 23%). IR: 3065w, 2965m, 2897s, 2845s, 2812m, 2727w, 2676w, 2653w, 1730w, 1599w, 1560m, 1477m, 1444s, 1374w, 1360m, 1354s, 1341w, 1313m, 1284s, 1275s, 1256s, 1251s, 1210w, 1184w, 1163w, 1134m, 1106s, 1082m, 1059m, 1046w, 1000w, 980w, 950m, 935m, 911w, 903w, 895w, 876w, 856m, 831m, 818m, 804m, 767w, 748w, 727w, 720w, 715w, 707w, 693w, 684w, 680w, 677w, 670w, 667w, 663w, 657w, 651w, 647w, 639w, 631w, 625w, 618w, 612w, 609w, 603w. UV-vis (THF) *λ*_max_ nm (*ε*, M^–1^ cm^–1^): 299 (19 500), 387 (4000 shoulder), 416 (4200), 480 (2000 shoulder), 600 (300). Anal. calcd for C_81_H_111_KN_2_NdO_9_: C, 67.56; H, 7.77; N, 1.95. Found: C, 66.23; H, 7.66; N, 1.71. The found CHN ratio of C_81_H_111.6_N_1.8_ is consistent with the formula and suggests incomplete combustion.

### [K(crypt)][((^Ad,Me^ArO)_3_mes)Gd] and [K(crypt)][((^Ad,Me^ArO)_3_mes)GdH], **2-Gd/3-Gd**

As described for **2-Nd**, [((^Ad,Me^ArO)_3_mes)Gd], **1-Gd**, (60 mg, 0.059 mmol) and 2.2.2-cryptand (23 mg, 0.060 mmol) were dissolved in 1 : 1 THF/C_6_H_6_ (4 mL) to form an off-white solution, which was combined with KC_8_ (20 mg, 0.15 mmol) to produce red crystals suitable for X-ray diffraction (51 mg). The crystals were characterized as a co-crystallized mixture of [K(crypt)][((^Ad,Me^ArO)_3_mes)Gd], **2-Gd**, and [K(crypt)][((^Ad,Me^ArO)_3_mes)GdH], **3-Gd**, of an approximate 65 : 35 ratio. UV-vis (THF) *λ*_max_ nm (*ε*, M^–1^ cm^–1^): 305 (22 000), 330 (6000 shoulder), 426 (4000), 520 (2000 shoulder), 580 (400).

### [K(crypt)][((^Ad,Me^ArO)_3_mes)Er] and [K(crypt)][((^Ad,Me^ArO)_3_mes)ErH], **2-Er/3-Er**

As described for **2-Nd**, [((^Ad,Me^ArO)_3_mes)Er], **1-Er**, (45 mg, 0.043 mmol) and 2.2.2-cryptand (16 mg, 0.043 mmol) were dissolved in 1 : 1 THF/C_6_H_6_ (2 mL) to form a pink solution, which was combined with KC_8_ (18 mg, 0.13 mmol) to produce red crystals suitable for X-ray diffraction (22 mg). The crystals were characterized as a cocrystallized mixture of [K(crypt)][((^Ad,Me^ArO)_3_mes)Er], **2-Er**, and [K(crypt)][((^Ad,Me^ArO)_3_mes)ErH], **3-Er**, of an approximate 55 : 45 ratio. UV-vis (THF) *λ*_max_ nm (*ε*, M^–1^ cm^–1^): 305 (21 000), 330 (4800 shoulder), 430 (5600), 500 (2500 shoulder), 600 (300).

### [K(crypt)][((^Ad,Me^ArO)_3_mes)Dy] and [K(crypt)][((^Ad,Me^ArO)_3_mes)DyH], **2-Dy/3-Dy**

[((^Ad,Me^ArO)_3_mes)Dy], **1-Dy**, (20 mg, 0.019 mmol) and 2.2.2-cryptand (7 mg, 0.02 mmol) were dissolved in THF (1 mL) to form a colorless solution. The solution was transferred to scintillation vial with a potassium smear (excess) and stored overnight at –35 °C. The resultant dark red solution was layered with Et_2_O (8 mL) and stored at –35 °C for 36 h to produce dark red crystals suitable for X-ray diffraction (10 mg). The crystals were characterized as a cocrystallized mixture of [K(crypt)][((^Ad,Me^ArO)_3_mes)Dy], **2-Dy**, and [K(crypt)][((^Ad,Me^ArO)_3_mes)DyH], **3-Dy**, of an approximate 63 : 37 ratio.

### [K(crypt)][((^Ad,Me^ArO)_3_mes)Dy] and [K(crypt)][((^Ad,Me^ArO)_3_mes)Dy(OH)], **2-Dy/4-Dy**

As described for **2-Nd**, [((^Ad,Me^ArO)_3_mes)Dy], **1-Dy**, (90 mg, 0.086 mmol) and 2.2.2-cryptand (32 mg, 0.085 mmol) were dissolved in 3 : 1 THF/C_6_H_6_ (3 mL) to form an off-white solution, which was combined with KC_8_ (18 mg, 0.13 mmol) to produce red crystals suitable for X-ray diffraction. The crystals were characterized as a co-crystallized mixture of [K(crypt)][((^Ad,Me^ArO)_3_mes)Dy], **2-Dy**, and [K(crypt)][((^Ad,Me^ArO)_3_mes)Dy(OH)], **4-Dy**, of an approximate 2 : 3 ratio.

### [K(18-crown-6)(THF)_2_][((^Ad,Me^ArO)_3_mes)Dy] and [K(18-crown-6)(THF)_2_][K(18-crown-6)(THF)_2_][((^Ad,Me^ArO)_3_mes)DyH], **5-Dy/6-Dy**

[((^Ad,Me^ArO)_3_mes)Dy], **1-Dy**, (50 mg, 0.048 mmol) and 18-crown-6 (13 mg, 0.048 mmol) were dissolved in THF (1 mL) to give a colorless solution. Excess potassium was added and the solution was stored overnight in the glovebox freezer. The resultant dark red solution was layered with Et_2_O (4 mL) and stored at –35 °C for 48 h at –35 °C to produce red crystals suitable for X-ray diffraction (29 mg). The crystals were characterized as a co-crystallized mixture of [K(18-crown-6)(THF)_2_][((^Ad,Me^ArO)_3_mes)Dy], **5-Dy**, and [K(18-crown-6)(THF)_2_][((^Ad,Me^ArO)_3_mes)DyH], **6-Dy**, of an approximate 1 : 1 ratio with two THF molecules in the lattice. UV-vis (THF) *λ*_max_ nm (*ε*, M^–1^ cm^–1^): 300 (18 000), 330 (3500 shoulder), 430 (4900), 480 (2600 shoulder), 550 (600 shoulder). Anal. calcd for C_91_H_131.5_DyKO_13_: C, 67.84; H, 8.20. Found: C, 65.15; H, 7.61. Additional elemental analyses experiments gave low carbon and hydrogen values. The found CH ratios of C_91_H_131_, C_91_H_129.7_ are consistent with the formula and suggest incomplete combustion.

### X-ray data collection, structure determination, and refinement

Crystallographic details for compounds **1-Ln** (Ln = Nd, Gd, Dy, and Er), **2-Nd**, **2-Ln/3-Ln** (Ln = Gd, Dy, Er), **2-Dy/4-Dy**, and **5-Dy/6-Dy** are summarized in the ESI.†

## Conflicts of interest

The authors declare no competing financial interest.
