# Supplementary material for: Comparisons of lanthanide/actinide +2 ions in a tris(aryloxide)arene coordination environment
Source: Chem Sci. 2017 Sep 7;8(11):7424–33. doi: 10.1039/c7sc02337e (PMC5674182; doi:10.1039/c7sc02337e)
Supplement: Supplementary file 1 [file SC-008-C7SC02337E-s001.pdf]

Electronic Supplementary Material (ESI) for Chemical Science.
This journal is © The Royal Society of Chemistry 2017

Comparisons of Lanthanide / Actinide +2 Ions in a Tris(aryloxide)arene Coordination Environment

Megan E. Fieser,[a] Chad T. Palumbo,[a] Henry S. La Pierre,[b] Dominik P. Halter,[b] Vamsee K. Voora,[a] Filipp Furche,*[a] Karsten Meyer,*[b] and William J. Evans*[a]

[a]Department of Chemistry, University of California, Irvine, California 92697-2025, United States

[b]Department of Chemistry and Pharmacy, Inorganic Chemistry, Friedrich-Alexander University Erlangen-Nürnberg (FAU), Egerlandstrasse 1, D-91058 Erlangen, Germany

Email: wevans@uci.edu, karsten.meyer@fau.de, filipp.furche@uci.edu

*To whom correspondence should be addressed.

Table of Contents

Crystallographic Details

Table S1	Crystal data and structure refinement for **1-Ln** (Ln = Nd, Gd, Dy, Er)	p. S3
Figure S1	Molecular structure of **1-Nd**	p. S4
Figure S2	Molecular structure of **1-Gd**	p. S5
Figure S3	Molecular structure of **1-Dy**	p. S6
Figure S4	Molecular structure of **1-Er**	p. S7
Table S2	Crystal data and structure refinement for **2-Nd** and **2-Ln/3-Ln** (Ln = Gd, Dy, Er)	p. S8
Figure S5	Molecular structure of **2-Nd**	p. S9
Figure S6	Molecular structure of **2-Gd/3-Gd**	p. S10
Figure S7	Molecular structure of **2-Dy/3-Dy**	p. S11
Figure S7	Molecular structure of **2-Er/3-Er**	p. S12
Table S3	Crystal data and structure refinement for **2-Dy/4-Dy** and **5-Dy/6-Dy**	p. S13
Figure S8	Molecular structure of **2-Dy/4-Dy**	p. S14
Figure S9	Molecular structure of **5-Dy/6-Dy**	p. S15
Table S4	Crystal data and structure refinement for $(^{Ad,Me}ArOH)_3mes$	p. S17
Figure S10	Molecular structure of $(^{Ad,Me}ArOH)_3mes$	p. S18

Spectroscopic Details

Figure S11	Electronic absorption spectra of $[(^{Ad,Me}ArO)_3mes]Ln$ (Ln = Nd, Gd)	p. S19
Figure S12	Experimental and simulated perpendicular mode X-band EPR spectra of **2-Gd/3-Gd** at 298 K in THF (1 mM)	p. S20
Figure S13	Experimental and simulated parallel mode X-band EPR spectra of **2-Gd/3-Gd** at 10 K in THF (1 mM)	p. S21
Figure S14	Experimental perpendicular mode X-band EPR spectra of **2-Gd/3-Gd** at 10 K in THF (1 mM)	p. S22
Figure S15	^{1}H NMR spectrum of **1-Nd** in C_6D_6 (~15 mM) at 298 K	p. S23
Figure S16	^{1}H NMR spectrum of **2-Nd** in THF-d_8 (~5 mM) at 298 K	p. S24

Computational Details p. S25

Figure S17	Isosurface for the lowest d-type unoccupied orbital **2-Nd** with a contour value of 0.05.	p. S26
Table S5.	Comparison of experimental and DFT calculated solution phase bond lengths and angles of of **2-Nd**, **1-Nd**, and **2-Gd** obtained from experimental crystal structures with those from DFT calculations in solution phase	p. S27
Table S6.	Mulliken population analysis of singly occupied orbitals of **2-Nd** and **2-Gd**.	p. S28
Table S7.	Mulliken atomic spin density analysis of **2-Nd** and **2-Gd**.	p. S28

References p. S29

Table S1. Crystal data and structure refinement for **1-Ln** (Ln = Nd, Gd, Dy, Er).

	**Nd**	**Gd**	**Dy**	**Er**
Empirical formula	$C_{63}H_{75}O_3Nd$	$C_{63}H_{75}O_3Gd$	$C_{63}H_{75}O_3Dy$	$C_{63}H_{75}O_3Er$
Formula weight	1024.47	1037.48	1042.73	1047.49
Temperature (K)	88(2)	88(2)	133(2)	133(2)
Space group	$P2_1/c$	$P2_1/c$	$P2_1/c$	$P2_1/c$
a (Å)	11.5124(10)	12.812(3)	12.7567(11)	12.7631(14)
b (Å)	36.810(3)	15.716(3)	15.6646(13)	15.6460(17)
c (Å)	11.9512(10)	29.962(6)	29.878(3)	29.910(3)
α (°)	90	90	90	90
β (°)	106.7298(9)	96.129(2)	96.2651(13)	96.4368(15)
γ (°)	90	90	90	90
Volume (Å^3)	4850.3(7)	5999(2)	5934.9(9)	5935.2(11)
Z	4	4	4	4
ρ_{calcd} (g/cm^3)	1.403	1.149	1.167	1.172
μ (mm^{-1})	1.119	1.145	1.299	1.454
$R1^a$	0.0453	0.0339	0.0444	0.0335
$wR2^b$	0.0850	0.0766	0.0943	0.0741

Definitions: $^a R1 = \sum||Fo| - |Fc||/\sum|Fo|$; $^b wR2 = [\sum[w(Fo^2 - Fc^2)^2]/ \sum[w(Fo^2)^2]]^{1/2}$.

[((^{Ad,Me}ArO)$_3$mes)Nd], 1-Nd. A blue crystal of approximate dimensions 0.301 x 0.088 x 0.071 mm was mounted on a glass fiber and transferred to a Bruker SMART APEX II diffractometer. The APEX2[1] program package was used to determine the unit-cell parameters and for data collection (120 sec/frame scan time for a sphere of diffraction data). The raw frame data was processed using SAINT[2] and SADABS[3] to yield the reflection data file. Subsequent calculations were carried out using the SHELXTL[4] program. The diffraction symmetry was $2/m$ and the systematic absences were consistent with the monoclinic space group $P2_1/c$ that was later determined to be correct.

The structure was solved using the coordinates from mef30, the La analog. The analytical scattering factors[5] for neutral atoms were used throughout the analysis. Hydrogen atoms were included using a riding model.

At convergence, wR2 = 0.0850 and Goof = 1.228 for 610 variables refined against 8875 data (0.83 Å), R1 = 0.0453 for those 7703 data with $I > 2.0\sigma(I)$.

Definitions:

$wR2 = [\Sigma[w(F_o^2-F_c^2)^2] / \Sigma[w(F_o^2)^2]]^{1/2}$

$R1 = \Sigma||F_o|-|F_c|| / \Sigma|F_o|$

$Goof = S = [\Sigma[w(F_o^2-F_c^2)^2] / (n-p)]^{1/2}$ where n is the number of reflections and p is the total number of parameters refined.

The thermal ellipsoid plot is shown at the 50% probability level.

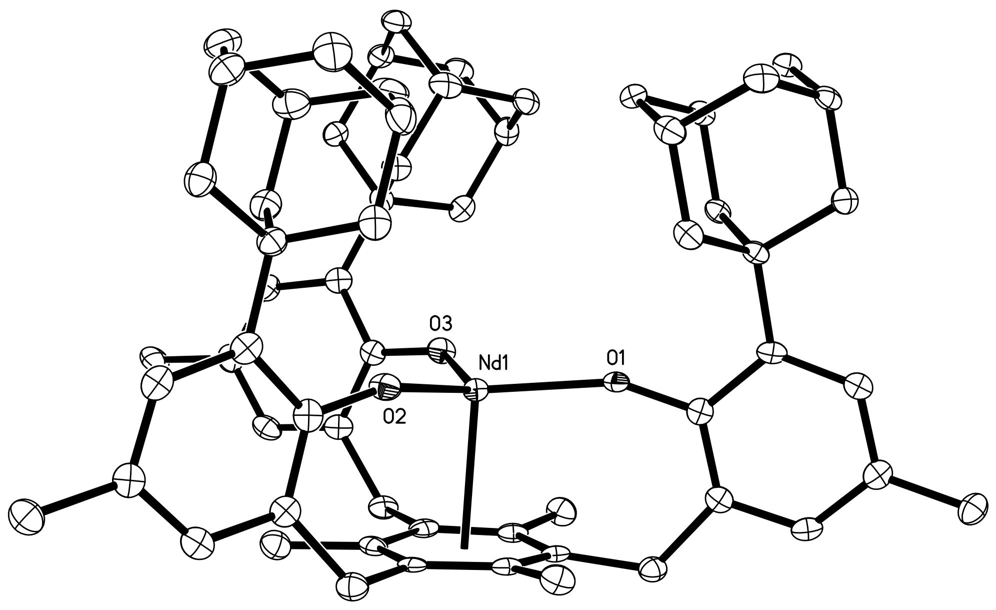

Figure S1. Molecular structure of [((^{Ad,Me}ArO)$_3$mes)Nd], **1-Nd**, with thermal ellipsoids drawn at the 50% probability level and hydrogen atoms omitted for clarity.

[((^{Ad,Me}ArO)$_3$mes)Gd], 1-Gd. A colorless crystal of approximate dimensions 0.031 x 0.062 x 0.330 mm was mounted in a cryoloop and transferred to a Bruker SMART APEX II diffractometer. The APEX2[1] program package was used to determine the unit-cell parameters and for data collection (90 sec/frame scan time for a sphere of diffraction data). The raw frame data was processed using SAINT[2] and SADABS[3] to yield the reflection data file. Subsequent calculations were carried out using the SHELXTL[4] program. The diffraction symmetry was $2/m$ and the systematic absences were consistent with the monoclinic space group $P2_1/c$ that was later determined to be correct.

The structure was solved by direct methods and refined on F^2 by full-matrix least-squares techniques. The analytical scattering factors[5] for neutral atoms were used throughout the analysis. Hydrogen atoms were included using a riding model.

At convergence, wR2 = 0.0766 and Goof = 1.014 for 610 variables refined against 12284 data (0.80Å), R1 = 0.0339 for those 9267 data with $I > 2.0\sigma(I)$.

There were several high residuals present in the final difference-Fourier map. It was not possible to determine the nature of the residuals although it was probable that diethyether or hexane solvents were present. The SQUEEZE[6a] routine in the PLATON[6b] program package was used to account for the electrons in the solvent accessible voids.

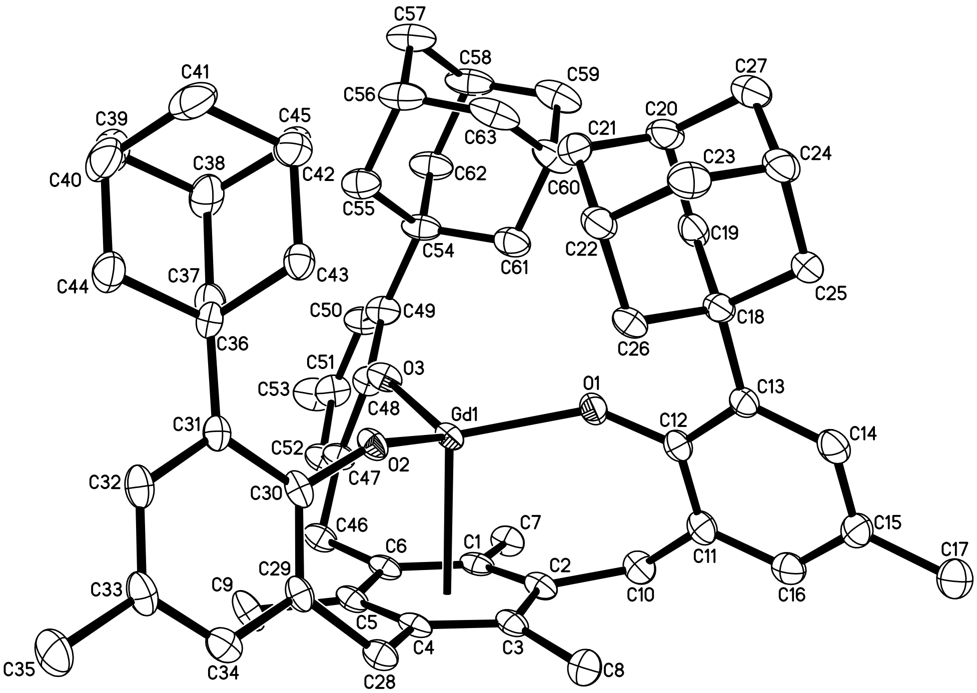

Figure S2. Molecular structure of [((^{Ad,Me}ArO)$_3$mes)Gd], **1-Gd**, with thermal ellipsoids drawn at the 50% probability level and hydrogen atoms omitted for clarity.

[((^{Ad,Me}ArO)$_3$mes)Dy], 1-Dy. A colorless crystal of approximate dimensions 0.030 x 0.0.050 x 0.100 mm was mounted in a cryoloop and transferred to a Bruker SMART APEX II diffractometer. The APEX2[1] program package was used to determine the unit-cell parameters and for data collection (90 sec/frame scan time for a sphere of diffraction data). The raw frame data was processed using SAINT[2] and SADABS[3] to yield the reflection data file. Subsequent calculations were carried out using the SHELXTL[4] program. The diffraction symmetry was $2/m$ and the systematic absences were consistent with the monoclinic space group $P2_1/c$ that was later determined to be correct.

The structure was solved by direct methods and refined on F^2 by full-matrix least-squares techniques. The analytical scattering factors[5] for neutral atoms were used throughout the analysis. Hydrogen atoms were included using a riding model.

At convergence, wR2 = 0.0943 and Goof = 0.958 for 610 variables refined against 12128 data (0.80Å), R1 = 0.0444 for those 8073 data with $I > 2.0\sigma(I)$.

There were several high residuals present in the final difference-Fourier map. It was not possible to determine the nature of the residuals although it was probable that diethyether or hexane solvents were present. The SQUEEZE[6a] routine in the PLATON[6b] program package was used to account for the electrons in the solvent accessible voids.

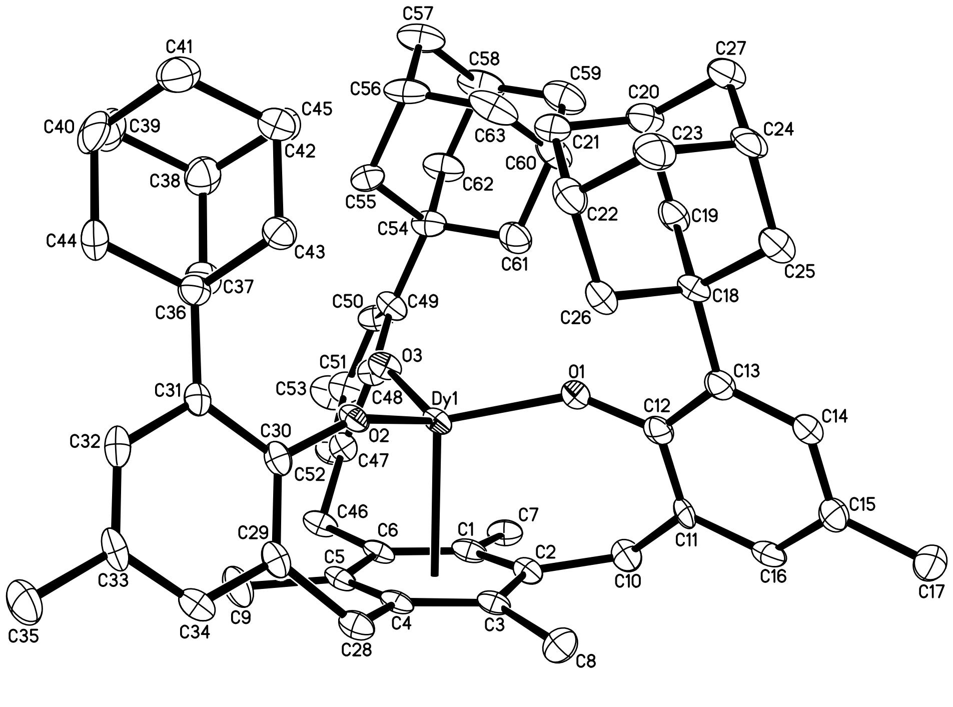

Figure S3. Molecular structure of [((^{Ad,Me}ArO)$_3$mes)Dy], **1-Dy**, with thermal ellipsoids drawn at the 50% probability level and hydrogen atoms omitted for clarity.

[((^{Ad,Me}ArO)$_3$mes)Er], 1-Er. A pink crystal of approximate dimensions 0.030 x 0.035 x 0.224 mm was mounted in a cryoloop and transferred to a Bruker SMART APEX II diffractometer. The APEX2[1] program package was used to determine the unit-cell parameters and for data collection (90 sec/frame scan time for a sphere of diffraction data). The raw frame data was processed using SAINT[2] and SADABS[3] to yield the reflection data file. Subsequent calculations were carried out using the SHELXTL[4] program. The diffraction symmetry was 2/*m* and the systematic absences were consistent with the monoclinic space group $P2_1/c$ that was later determined to be correct.

The structure was solved by direct methods and refined on F^2 by full-matrix least-squares techniques. The analytical scattering factors[5] for neutral atoms were used throughout the analysis. Hydrogen atoms were included using a riding model.

At convergence, wR2 = 0.0741 and Goof = 0.985 for 610 variables refined against 12175 data (0.80Å), R1 = 0.0335 for those 9178 data with $I > 2.0\sigma(I)$.

There were several high residuals present in the final difference-Fourier map. It was not possible to determine the nature of the residuals although it was probable that diethyether or hexane solvents were present. The SQUEEZE[6a] routine in the PLATON[6b] program package was used to account for the electrons in the solvent accessible voids.

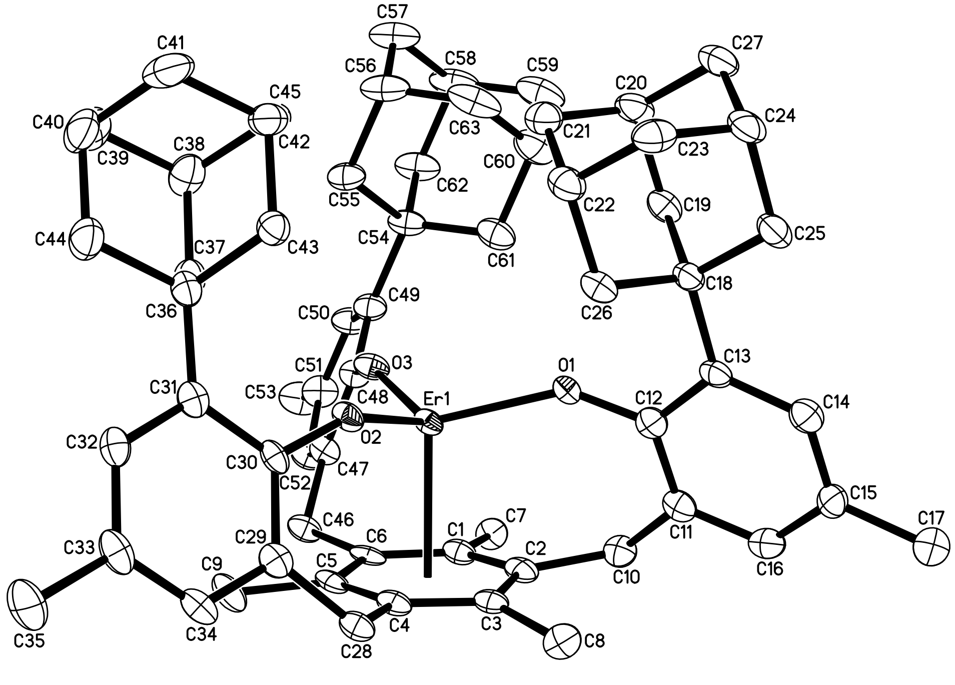

Figure S4. Molecular structure of [((^{Ad,Me}ArO)$_3$mes)Er], **1-Er**, with thermal ellipsoids drawn at the 50% probability level and hydrogen atoms omitted for clarity.

Table S2. Crystal data and structure refinement for **2-Nd**, and **2-Ln/3-Ln** (Ln = Gd, Dy, Er,).

	**2-Nd**	**2-Gd/3-Gd**	**2-Dy/3-Dy**	**2-Er/3-Er**
Empirical formula	$C_{81}H_{111}KN_2NdO_9$	$C_{81}H_{111}GdKN_2O_9$ $(H)_{0.35} \cdot (C_4H_{10}O)$	$C_{81}H_{111}DyKN_2O_9$ $H_{0.37} \cdot (C_4H_{10}O)$	C_{81} H_{111} Er K N_2 $O_9 (H)_{0.45} \cdot (C_4H_{10}O)$
Formula weight	1440.05	1527.53	1532.80	1537.64
Temperature (K)	88(2)	88(2)	133(2)	100(2)
Space group	$P2_13$	$P2_13$	$P2_13$	$P2_13$
a (Å)	19.7666(8)	19.7291(9)	19.798(3)	19.7501(6)
b (Å)	19.7666(8)	19.7291(9)	19.798(3)	19.7501(6)
c (Å)	19.7666(8)	19.7291(9)	19.798(3)	19.7501(6)
α (°)	90	90	90	90
β (°)	90	90	90	90
γ (°)	90	90	90	90
Volume (Å^3)	7723.2(9)	7679.3(11)	7760(3)	7703.9(7)
Z	4	4	4	4
ρ_{calcd} (g/cm^3)	1.238	1.321	1.312	1.326
μ (mm^{-1})	0.781	0.978	1.076	1.203
$R1^a$	0.0482	0.0372	0.0417	0.0413
$wR2^b$	0.1279	0.0951	0.0970	0.0827

Definitions: $^a R1 = \sum||Fo| - |Fc||/\sum|Fo|$; $^b wR2 = [\sum[w(Fo^2 - Fc^2)^2]/ \sum[w(Fo^2)^2]]^{1/2}$.

[K(crypt)][((^{Ad,Me}ArO)$_3$mes)Nd], 2-Nd. A red crystal of approximate dimensions 0.349 x 0.234 x 0.214 mm was mounted on a glass fiber and transferred to a Bruker SMART APEX II diffractometer. The APEX2[7] program package was used to determine the unit-cell parameters and for data collection (20 sec/frame scan time for a sphere of diffraction data). The raw frame data was processed using SAINT[2] and SADABS[8] to yield the reflection data file. Subsequent calculations were carried out using the SHELXTL[9] program. The diffraction symmetry was $m\bar{3}$ and the systematic absences were consistent with the cubic space group $P2_13$ that was later determined to be correct.

The structure was solved by direct methods and refined on F^2 by full-matrix least-squares techniques. The analytical scattering factors[5] for neutral atoms were used throughout the analysis. Hydrogen atoms were included using a riding model. The molecule was located on a three-fold rotation axis.

At convergence, wR2 = 0.1279 and Goof = 1.174 for 285 variables refined against 6739 data (0.73 Å), R1 = 0.0482 for those 6400 data with $I > 2.0\sigma(I)$. The absolute structure was assigned by refinement of the Flack parameter.[10]

There were several high residuals present in the final difference-Fourier map. It was not possible to determine the nature of the residuals although it was probable that toluene solvent was present. The SQUEEZE routine in the PLATON[6b] program package was used to account for the electrons in the solvent accessible voids

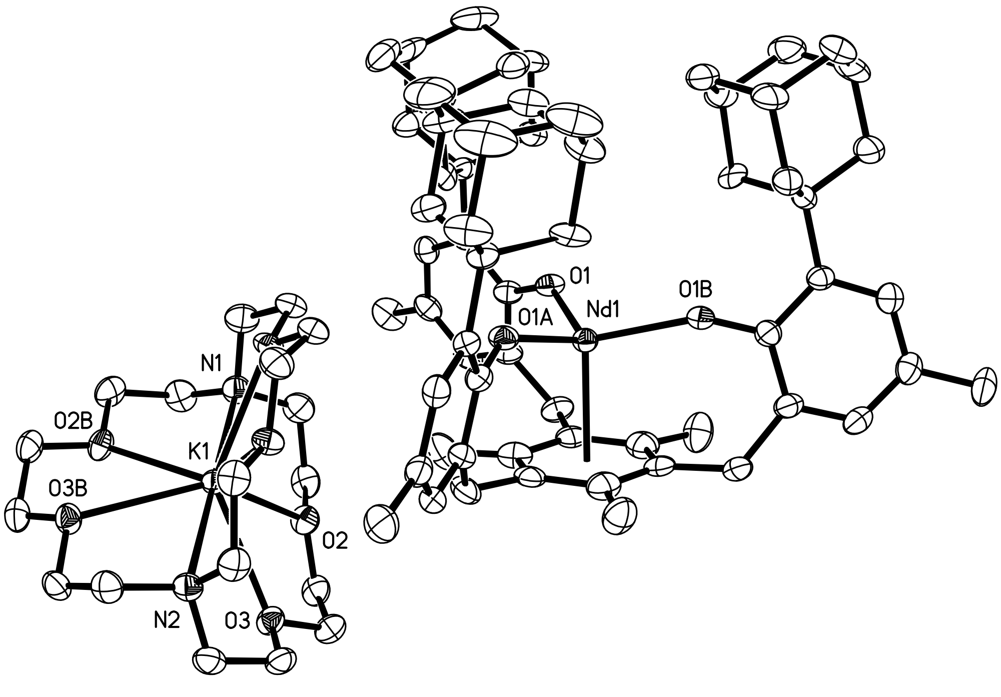

Figure S5. Molecular structure of [K(crypt)][((^{Ad,Me}ArO)$_3$mes)Nd], **2-Nd**, with thermal ellipsoids drawn at the 50% probability level and hydrogen atoms omitted for clarity.

[K(2.2.2-cryptand)]{[(^{Ad,Me}ArO)$_3$mes]Gd} and [K(2.2.2-cryptand)]{[(^{Ad,Me}ArO)$_3$mes]GdH}, 2-Gd/3-Gd. A red crystal of approximate dimensions 0.102 x 0.177 x 0.181 mm was mounted on a glass fiber and transferred to a Bruker SMART APEX II diffractometer. The APEX2[11] program package was used to determine the unit-cell parameters and for data collection (180 sec/frame scan time for a sphere of diffraction data). The raw frame data was processed using SAINT[2] and SADABS[8] to yield the reflection data file. Subsequent calculations were carried out using the SHELXTL[4] program. The systematic absences were consistent with the cubic space group $P2_13$ that was later determined to be correct.

The structure was solved by direct methods and refined on F^2 by full-matrix least-squares techniques. The analytical scattering factors[5] for neutral atoms were used throughout the analysis. Hydrogen atoms were included using a riding model. The molecule and counter-ion were located on three-fold rotation axes. The complex appeared to be a mixed composition of approximately Gd^{2+} (65%) / Gd^{3+}(35%). There was approximately 35% of a hydride ligand present bound to Gd(2). The position of the hydride was evident in the electron density map however it was necessary to fix the Gd-H distance and the hydride thermal parameter during refinement. An ether solvent molecule was disordered about a three-fold rotation axis and included with partial site-occupancy-factors.

At convergence, wR2 = 0.0951 and Goof = 1.222 for 301 variables refined against 5062 data (0.81), R1 = 0.0372 for those 4861 data with $I > 2.0\sigma(I)$. The absolute structure was assigned by refinement of the Flack parameter.[10]

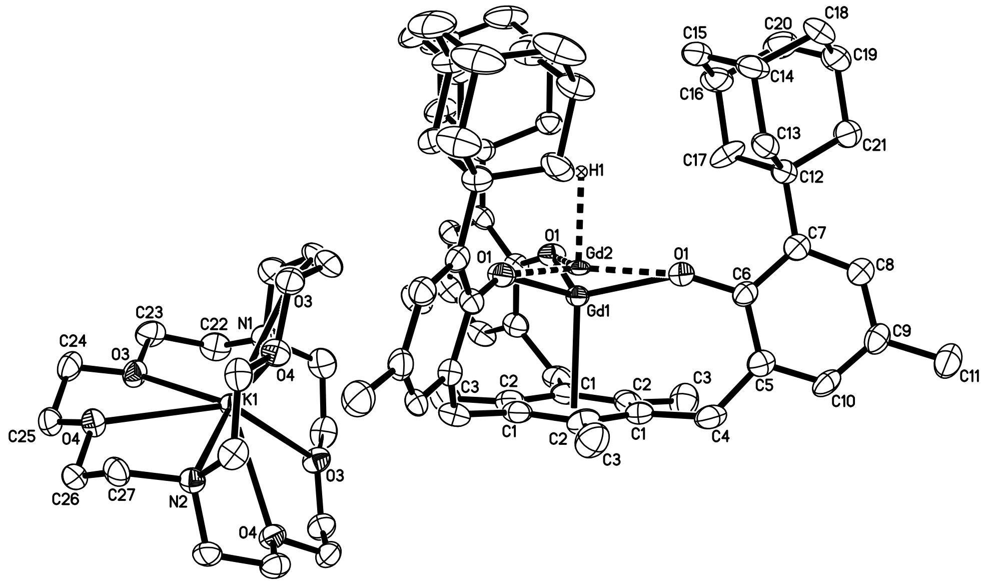

Figure S6. Molecular structure of [K(2.2.2-cryptand)]{[(^{Ad,Me}ArO)$_3$mes]Gd} and [K(2.2.2-cryptand)] {[(^{Ad,Me}ArO)$_3$mes]GdH}, **2-Gd/3-Gd**, with thermal ellipsoids drawn at the 50% probability level and hydrogen atoms omitted for clarity.

[K(2.2.2-cryptand)]{[(^{Ad,Me}ArO)$_3$mes]Dy} and [K(2.2.2-cryptand)]{[(^{Ad,Me}ArO)$_3$mes]DyH}, 2-Dy/3-Dy.

A red crystal of approximate dimensions 0.371 x 0.438 x 0.566 mm was mounted in a cryoloop and transferred to a Bruker SMART APEX II diffractometer. The APEX2[1] program package was used to determine the unit-cell parameters and for data collection (10 sec/frame scan time for a sphere of diffraction data). The raw frame data was processed using SAINT[2] and SADABS[3] to yield the reflection data file. Subsequent calculations were carried out using the SHELXTL[4] program. The systematic absences were consistent with the cubic space group $P2_13$ that was later determined to be correct.

The structure was solved by dual space methods and refined on F^2 by full-matrix least-squares techniques. The analytical scattering factors[5] for neutral atoms were used throughout the analysis. Hydrogen atoms were included using a riding model. The molecule and counter-ion were located on three-fold rotation axes. The complex appeared to be a mixed composition of approximately Dy^{2+} (63%) / Dy^{3+}(37%). There was approximately 37% hydride ligand present bound to Dy(2). The position of the hydride was located from difference map however it was necessary to fix the Dy-H distance and the hydride thermal parameter during refinement. An ether solvent molecule was disordered about a three-fold rotation axis and included with partial site-occupancy-factors.

At convergence, wR2 = 0.0970 and Goof = 1.218 for 307 variables refined against 6642 data (0.74), R1 = 0.0417 for those 6365 data with I > 2.0σ(I). The absolute structure was assigned by refinement of the Flack parameter.[10] The structure was refined as a two-component twin.

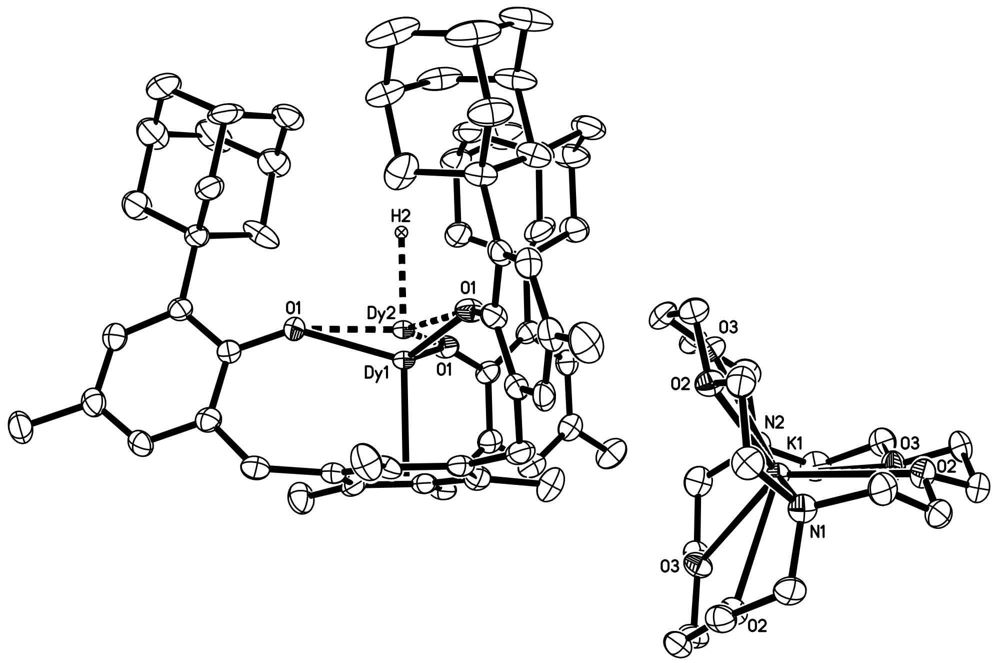

Figure S7. Molecular structure of [K(crypt)][((^{Ad,Me}ArO)$_3$mes)Dy] and [K(crypt)][((^{Ad,Me}ArO)$_3$mes)DyH], **2-Dy/3-Dy**, with thermal ellipsoids drawn at the 50% probability level and hydrogen atoms omitted for clarity.**[K(crypt)][((^{Ad,Me}ArO)$_3$mes)Er] and [K(crypt)][((^{Ad,Me}ArO)$_3$mes)ErH], 2-Er/3-Er.** A red crystal of approximate dimensions 0.200 x 0.240 x 0.280 mm was mounted on a glass fiber and transferred to a Bruker

SMART APEX II diffractometer. The APEX2[11] program package was used to determine the unit-cell parameters and for data collection (3 sec/frame scan time for 99 frames of diffraction data). The raw frame data was processed using SAINT[2] and SADABS[3] to yield the reflection data file. Subsequent calculations were carried out using the SHELXTL[4] program. The systematic absences were consistent with the cubic space group $P2_13$ that was later determined to be correct.

The structure was solved by direct methods and refined on F^2 by full-matrix least-squares techniques. The analytical scattering factors[5] for neutral atoms were used throughout the analysis. Hydrogen atoms were included using a riding model. The molecule and counter-ion were located on three-fold rotation axes. The complex appeared to be a mixed composition of approximately Er^{2+} (55%) / Er^{3+}(45%). There was approximately 45% of a hydride ligand present bound to Er(2). The position of the hydride was evident in the electron density map however it was necessary to fix the Er-H distance and the hydride thermal parameter during refinement. An ether solvent molecule was disordered about a three-fold rotation axis and included with partial site-occupancy-factors.

At convergence, wR2 = 0.0827 and Goof = 1.114 for 301 variables refined against 5953 data (0.73), R1 = 0.0413 for those 5352 data with $I > 2.0\sigma(I)$. The absolute structure was assigned by refinement of the Flack parameter[10].

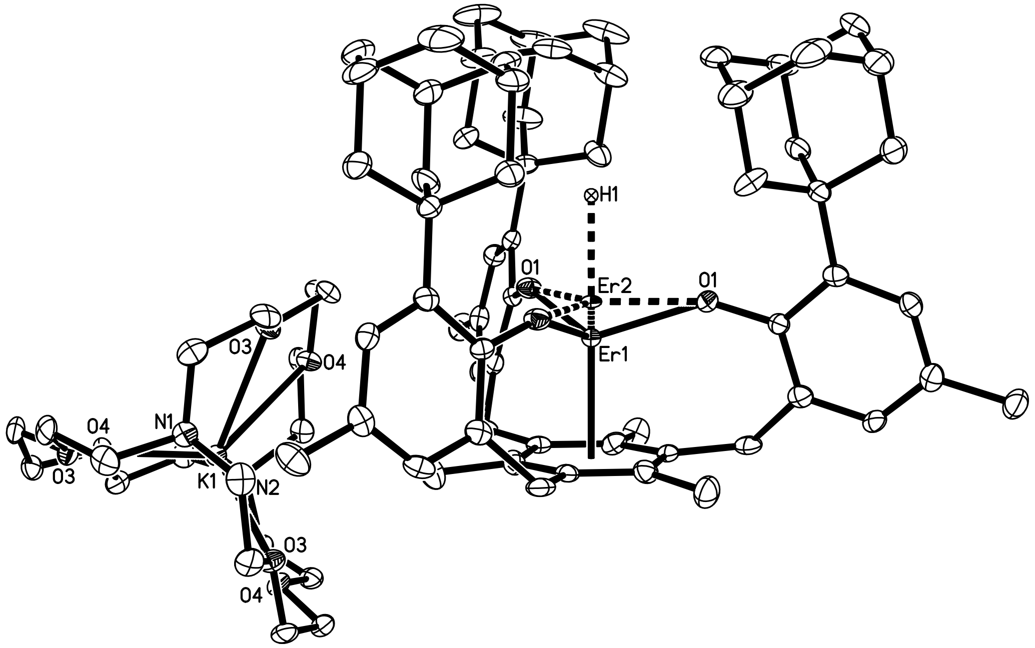

Figure S7. Molecular structure of [K(crypt)][((^{Ad,Me}ArO)$_3$mes)Er] and [K(crypt)][((^{Ad,Me}ArO)$_3$mes)ErH], **2-Er/3-Er**, with thermal ellipsoids drawn at the 50% probability level and hydrogen atoms omitted for clarity.**Table S3.** Crystal data and structure refinement for **2-Dy/4-Dy** and **5-Dy/6-Dy**.

	**2-Dy/4-Dy**	**5-Dy/6-Dy**

Empirical formula	$C_{81}H_{111}DyKN_2O_{10}(OH)_{0.6} \cdot (C_4H_{10}O)$	$C_{83}H_{115.5}DyKO_{11} \bullet 2(C_4H_8O)$
Formula weight	1542.64	1635.05
Temperature (K)	100(2)	133(2)
Space group	$P2_13$	*Pbcn*
a (Å)	19.7730(7)	21.7362(14)
b (Å)	19.7730(7)	30.6130(19)
c (Å)	19.7730(7)	25.4472(16)
α (°)	90	90
β (°)	90	90
γ (°)	90	90
Volume (Å^3)	7730.7(8)	16932.8(19)
Z	4	8
ρ_{calcd} (g/cm^3)	1.325	1.283
μ (mm^{-1})	1.081	0.992
$R1^a$	0.0457	0.0583
$wR2^b$	0.0956	0.1486

Definitions: $^a R1 = \sum||Fo| - |Fc||/\sum|Fo|$; $^b wR2 = [\sum[w(Fo^2 - Fc^2)^2]/ \sum[w(Fo^2)^2]]^{1/2}$.

[K(crypt)][((^{Ad,Me}ArO)$_3$mes)Dy] and [K(crypt)][((^{Ad,Me}ArO)$_3$mes)Dy(OH)], 2-Dy/4-Dy. A red crystal of approximate dimensions 0.200 x 0.230 x 0.260 mm was mounted on a glass fiber and transferred to a Bruker SMART APEX II diffractometer. The APEX2[11] program package was used to determine the unit-cell parameters and for data collection (10 sec/frame scan time for 112 frames of data). The raw frame data was processed using SAINT[2] and SADABS[3] to yield the reflection data file. Subsequent calculations were carried out using the SHELXTL[4] program. The systematic absences were consistent with the cubic space group $P2_13$ that was later determined to be correct.

The structure was solved by direct methods and refined on F^2 by full-matrix least-squares techniques. The analytical scattering factors[5] for neutral atoms were used throughout the analysis. Hydrogen atoms were included using a riding model. The molecule and counter-ion were located on three-fold rotation axes. The complex appeared to be a mixed composition of approximately Dy^{2+} (40%) / Dy^{3+}(60%). There was approximately 60% of a hydroxide ligand present. An ether solvent molecule was disordered about a three-fold rotation axis and included with partial site-occupancy-factors.

At convergence, wR2 = 0.0956 and Goof = 1.043 for 303 variables refined against 4155 data (0.83Å), R1 = 0.0457 for those 3582 data with $I > 2.0\sigma(I)$. The absolute structure was assigned by refinement of the Flack parameter[10].

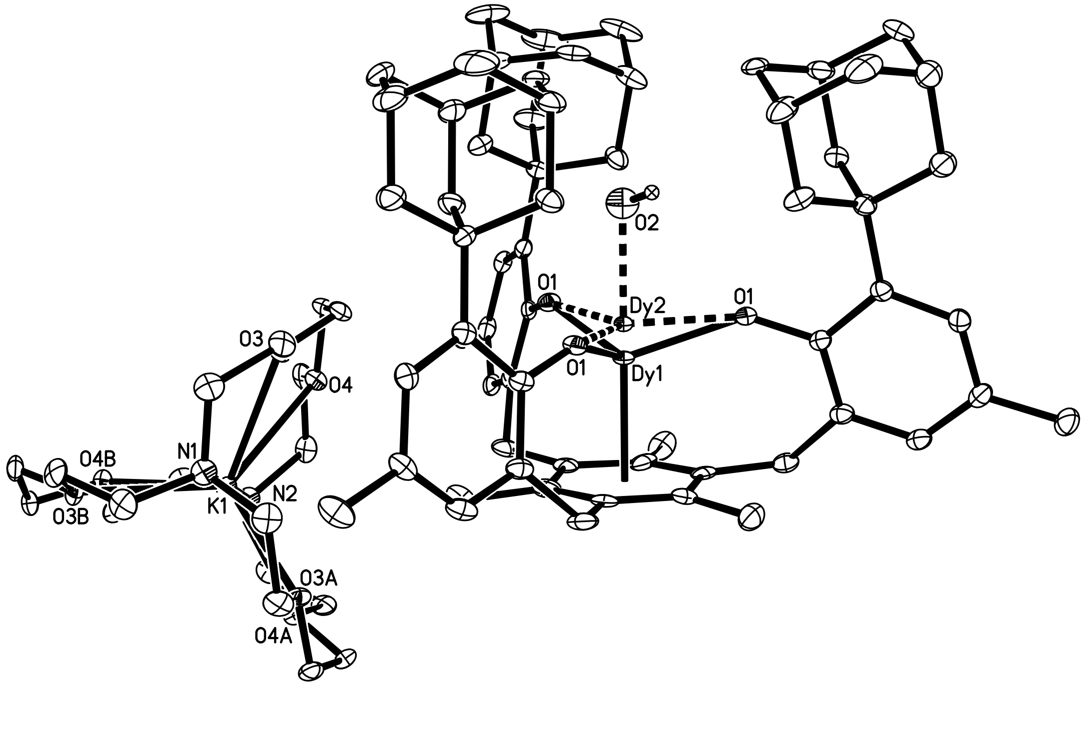

Figure S8. Molecular structure of [K(crypt)][((^{Ad,Me}ArO)$_3$mes)Dy] and [K(crypt)][((^{Ad,Me}ArO)$_3$mes)Dy(OH)], **2-Dy/4-Dy**, with thermal ellipsoids drawn at the 50% probability level and hydrogen atoms omitted for clarity.

[K(18-crown-6)(THF)$_2$][((^{Ad,Me}ArO)$_3$mes)Dy] and [K(18-crown-6)(THF)$_2$][K(18-crown-6)(THF)$_2$][((^{Ad,Me}ArO)$_3$mes)DyH], 5-Dy/6-Dy. A brown crystal of approximate dimensions 0.128 x 0.194 x 0.231 mm was mounted in a cryoloop and transferred to a Bruker SMART APEX II diffractometer. The APEX2[1] program package was used to determine the unit-cell parameters and for data collection (90 sec/frame scan time for a sphere of diffraction data). The raw frame data was processed using SAINT[2] and SADABS[3] to yield the reflection data file. Subsequent calculations were carried out using the SHELXTL[4] program. The diffraction symmetry was *mmm* and the systematic absences were consistent with the orthorhombic space group *Pbcn* that was later determined to be correct.

The structure was solved by direct methods and refined on F^2 by full-matrix least-squares techniques. The analytical scattering factors[5] for neutral atoms were used throughout the analysis. The complex appeared to be a mixed composition of approximately Dy^{2+} (50%) / Dy^{3+}(50%). There was approximately 50% of a hydride ligand present which was located from a difference-Fourier map and refined (x,y,z and U_{iso}). Hydrogen atoms were included using a riding model. There were two molecules of tetrahydrofuran solvent present.

Least-squares analysis yielded wR2 = 0.1486 and Goof = 1.101 for 949 variables refined against 16156 data (0.82Å), R1 = 0.0583 for those 12496 data with $I > 2.0\sigma(I)$.

There were several high residuals present in the final difference-Fourier map. It was not possible to determine the nature of the residuals although it was probable based on the observed geometry that diethylether solvent was present. The SQUEEZE[6a] routine in the PLATON[6b] program package was used to account for the electrons in the solvent accessible voids.

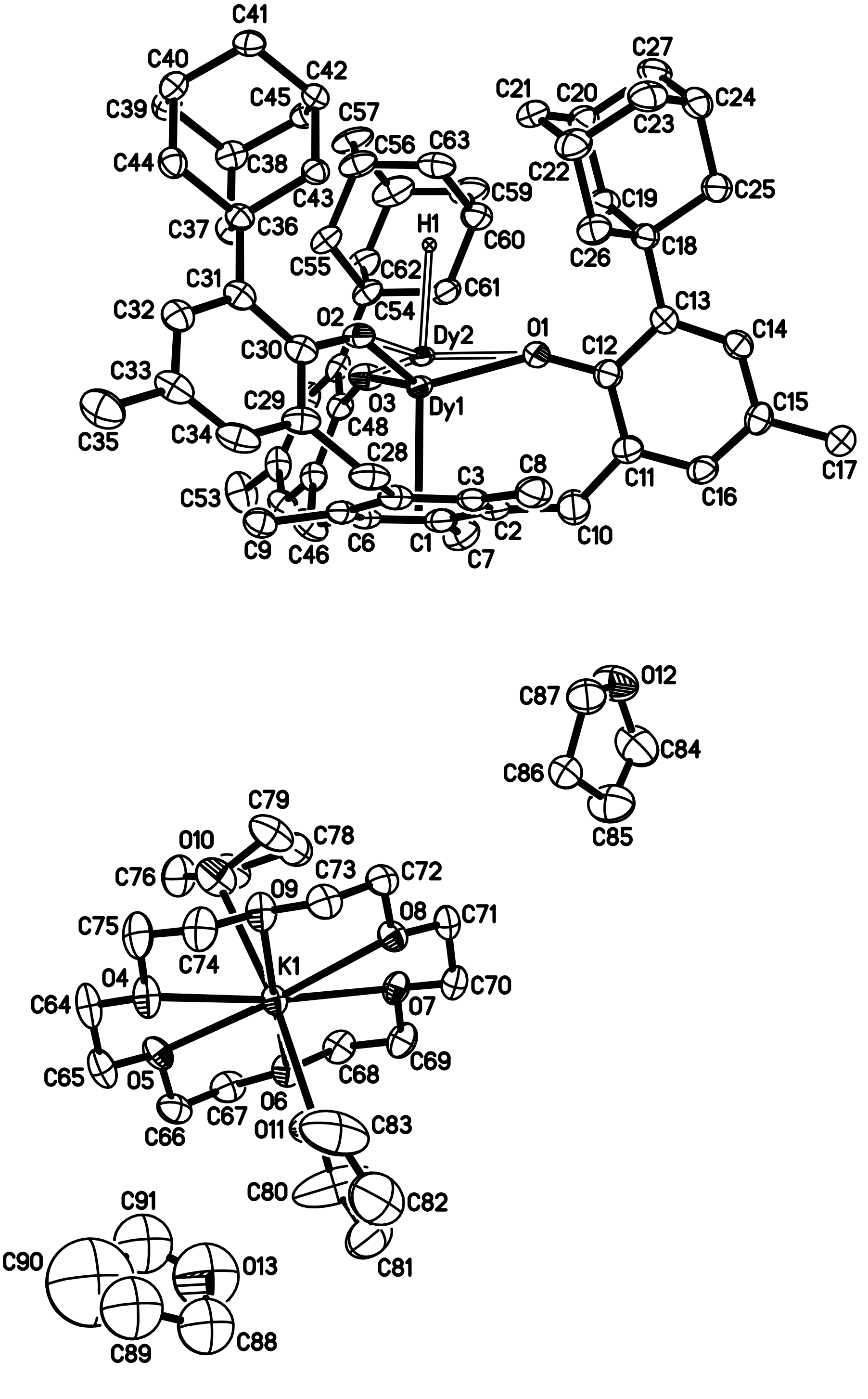

Figure S9. Molecular structure of [K(18-crown-6)(THF)$_2$][((^{Ad,Me}ArO)$_3$mes)Dy] and [K(18-crown-6)(THF)$_2$][K(18-crown-6)(THF)$_2$][((^{Ad,Me}ArO)$_3$mes)DyH], **5-Dy/6-Dy**, with thermal ellipsoids drawn at the 50% probability level and hydrogen atoms omitted for clarity.

Table S4. Crystal data and structure refinement for $(^{Ad,Me}ArOH)_3mes$.

	$(^{Ad,Me}ArOH)_3mes$
Empirical formula	$C_{63}H_{78}O_3H_3 \cdot 3(C_4H_{10}O)$
Formula weight	1105.61
Temperature (K)	133(2)
Space group	$P\bar{1}$
a (Å)	13.904(3)
b (Å)	15.774(3)
c (Å)	16.856(3)
α (°)	99.238(3)
β (°)	109.444(2)
γ (°)	106.032(3)
Volume (Å^3)	3217.6(11)
Z	2
ρ_{calcd} (g/cm^3)	1.141
μ (mm^{-1})	0.070
$R1^a$	0.0570
$wR2^b$	0.1517

Definitions: $^a R1 = \sum||Fo| - |Fc||/\sum|Fo|$; $^b wR2 = [\sum[w(Fo^2 - Fc^2)^2]/ \sum[w(Fo^2)^2]]^{1/2}$.

(^{Ad,Me}ArOH)$_3$mes. A colorless crystal of approximate dimensions 0.172 x 0.200 x 0.292 mm was mounted in a cryoloop and transferred to a Bruker SMART APEX II diffractometer. The APEX2[1] program package was used to determine the unit-cell parameters and for data collection (90 sec/frame scan time for a sphere of diffraction data). The raw frame data was processed using SAINT[2] and SADABS[3] to yield the reflection data file. Subsequent calculations were carried out using the SHELXTL[4] program. There were no systematic absences nor any diffraction symmetry other than the Friedel condition. The centrosymmetric triclinic space group $P\bar{1}$ was assigned and later determined to be correct.

The structure was solved by direct methods and refined on F^2 by full-matrix least-squares techniques. The analytical scattering factors[5] for neutral atoms were used throughout the analysis. Hydrogen atoms H(1), H(2) and H(3) were located from a difference-Fourier map and refined (x,y,z and U_{iso}). The remaining hydrogen atoms were included using a riding model. There were three molecules of diethylether present.

Least-squares analysis yielded wR2 = 0.1517 and Goof = 0.96 for 754 variables refined against 11758 data (0.83 Å), R1 = 0.0570 for those 7122 data with I > 2.0σ(I).

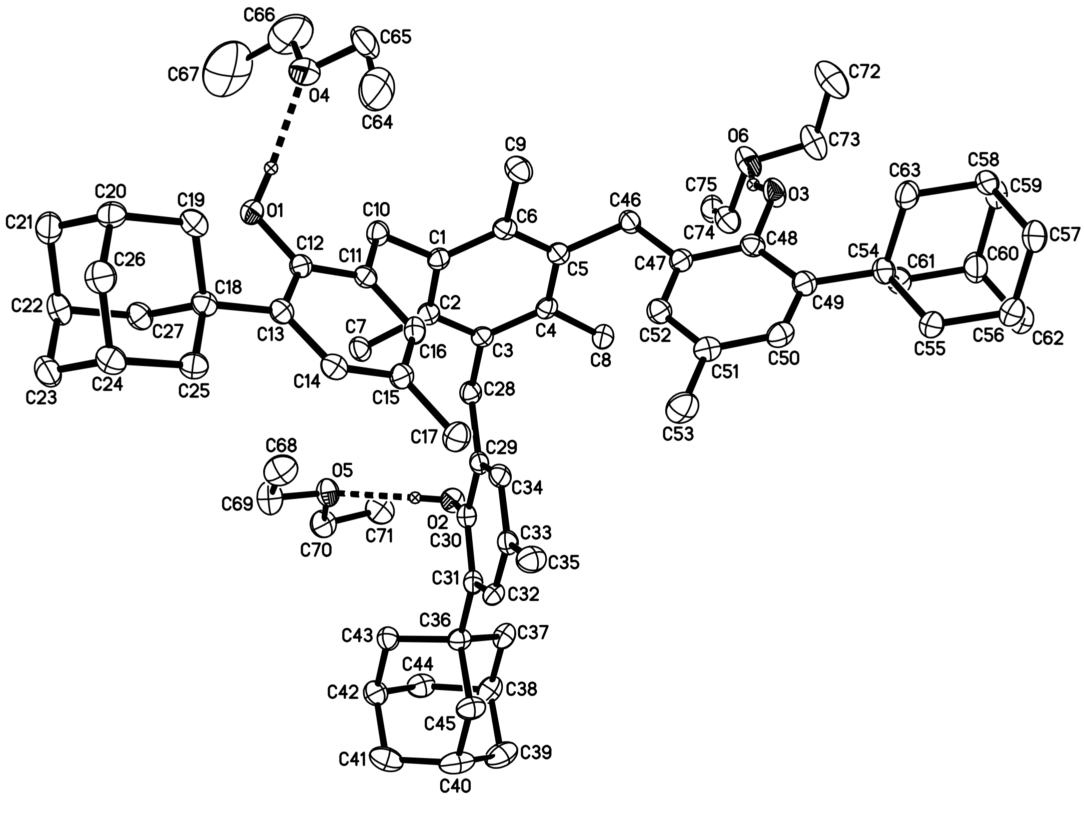

Figure S10. Molecular structure of (^{Ad,Me}ArOH)$_3$mes. with thermal ellipsoids drawn at the 50% probability level and hydrogen atoms omitted for clarity.

Spectroscopic Details

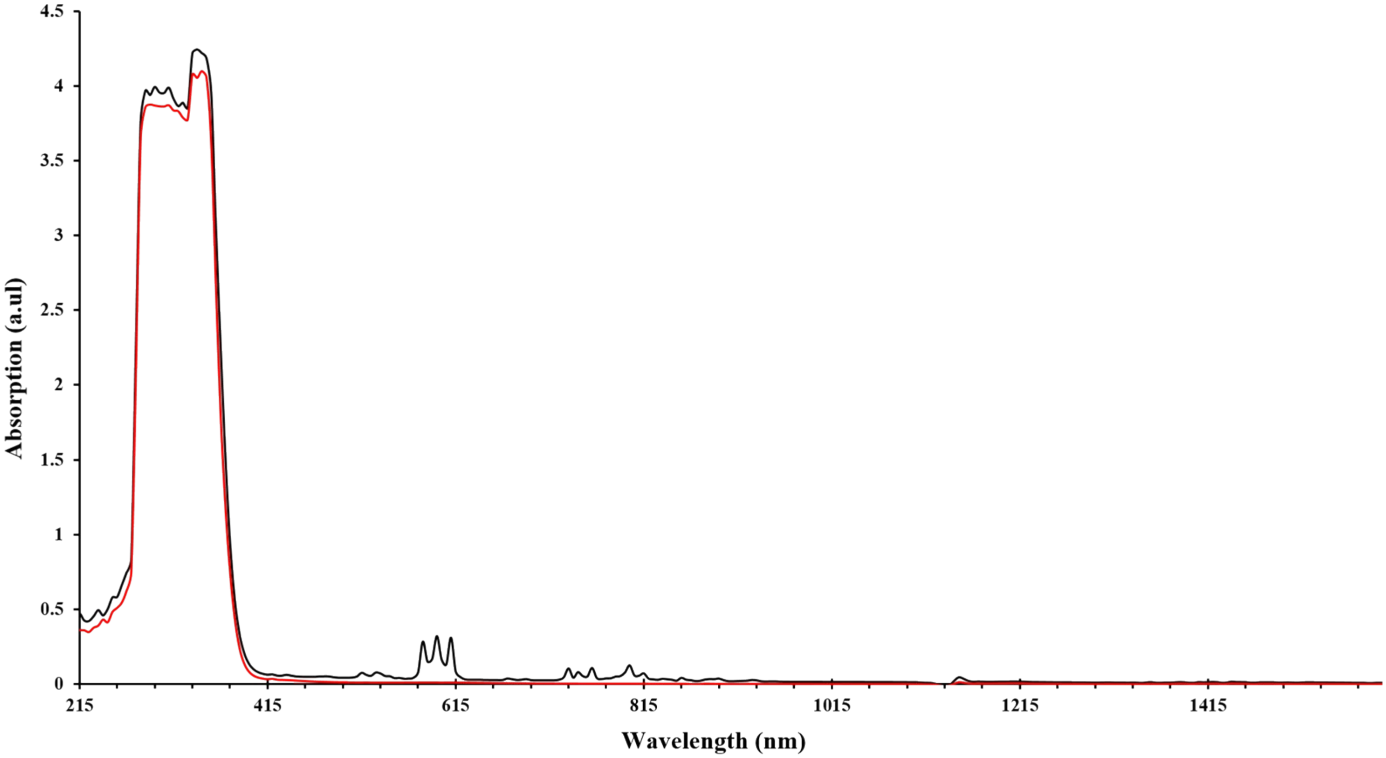

Figure S11. Electronic absorption spectra of [(^{Ad,Me}ArO)$_3$mes]Ln, **1-Ln** (Ln = Nd (black), Gd (red), as 10 mM solutions in benzene at room temperature.

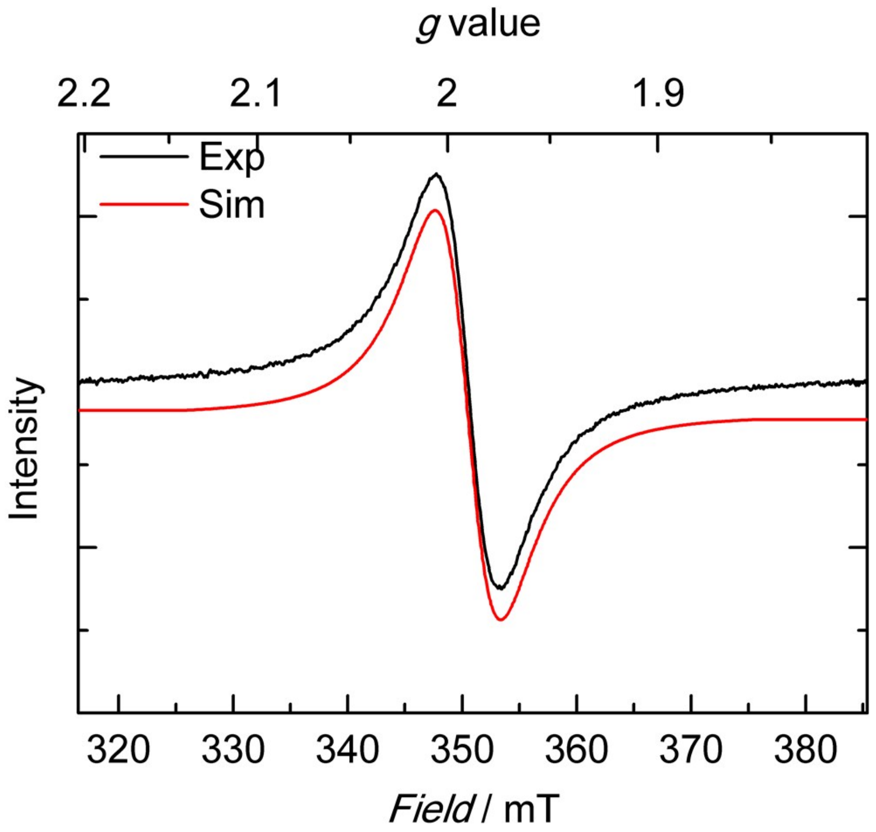

Figure S12. Experimental (black) and simulated (red) X-band EPR spectra of single crystals of **2-Gd/3-Gd** dissolved in THF (1 mM) at 298 K (perpendicular mode; g_{iso} = 1.990; ν = 9.762 GHz; P = 0.0203 mW; modulation amplitude = 0.902 mT). The simulation performed using W95EPR[12] gave g_{iso} = 1.990 and W_{iso} = 4.96 mT.

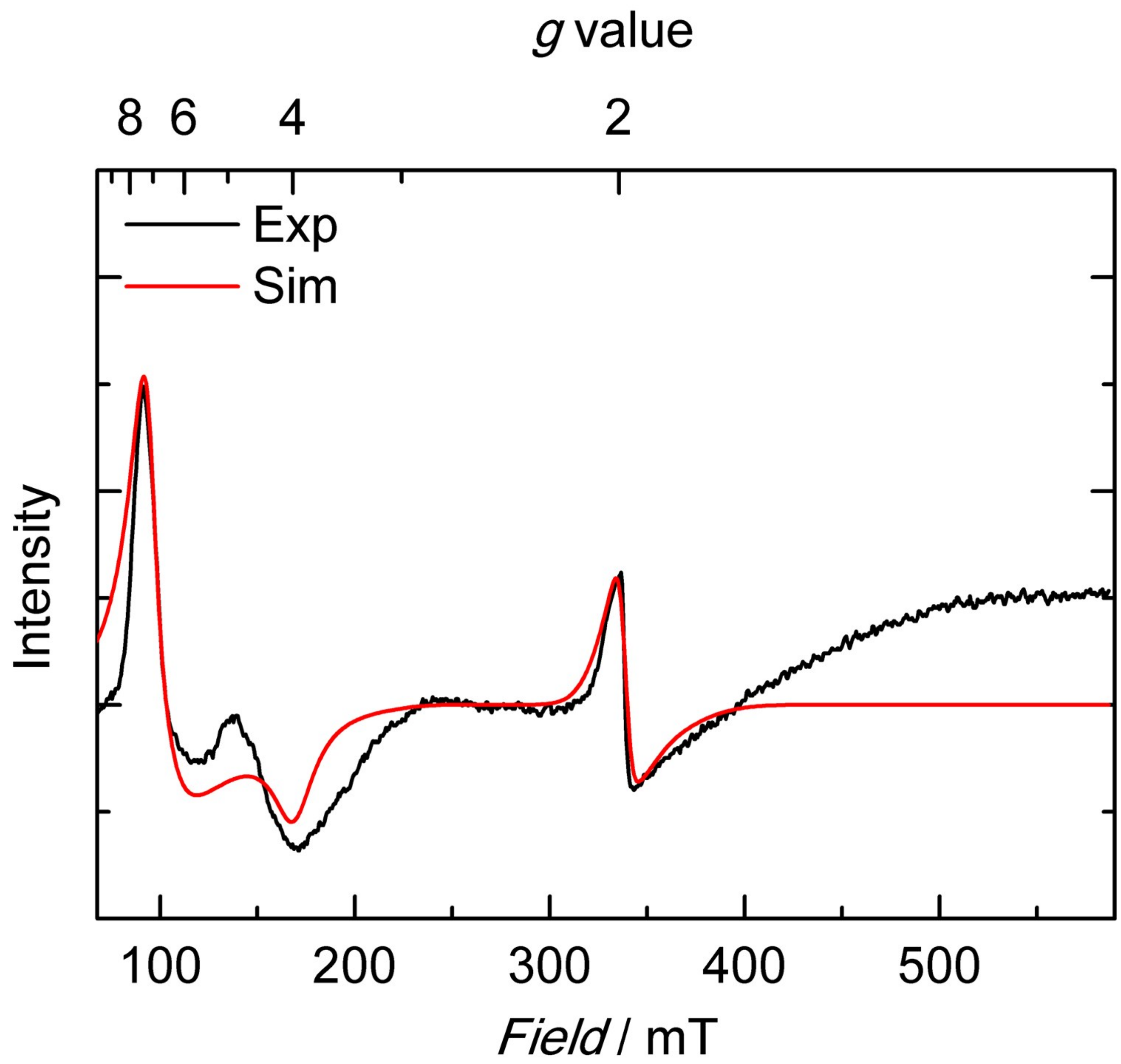

Figure S13. Experimental (black) and simulated (red) parallel mode X-band EPR spectra of single crystals of **2-Gd/3-Gd** dissolved in THF (1 mM) at 10 K. The spectrum was simulated[12] with two species (Gd(III) 10%, Gd(II) 90%) Gd(II): g_1 = 7.02, g_2 = 6.95, g_3 = 3.97, Gd(III): g_1 = 1.987, g_2 = 1.976, g_3 = 1.847; ν = 9.383 GHz; P = 2.026 mW; modulation amplitude = 1.002 mT).

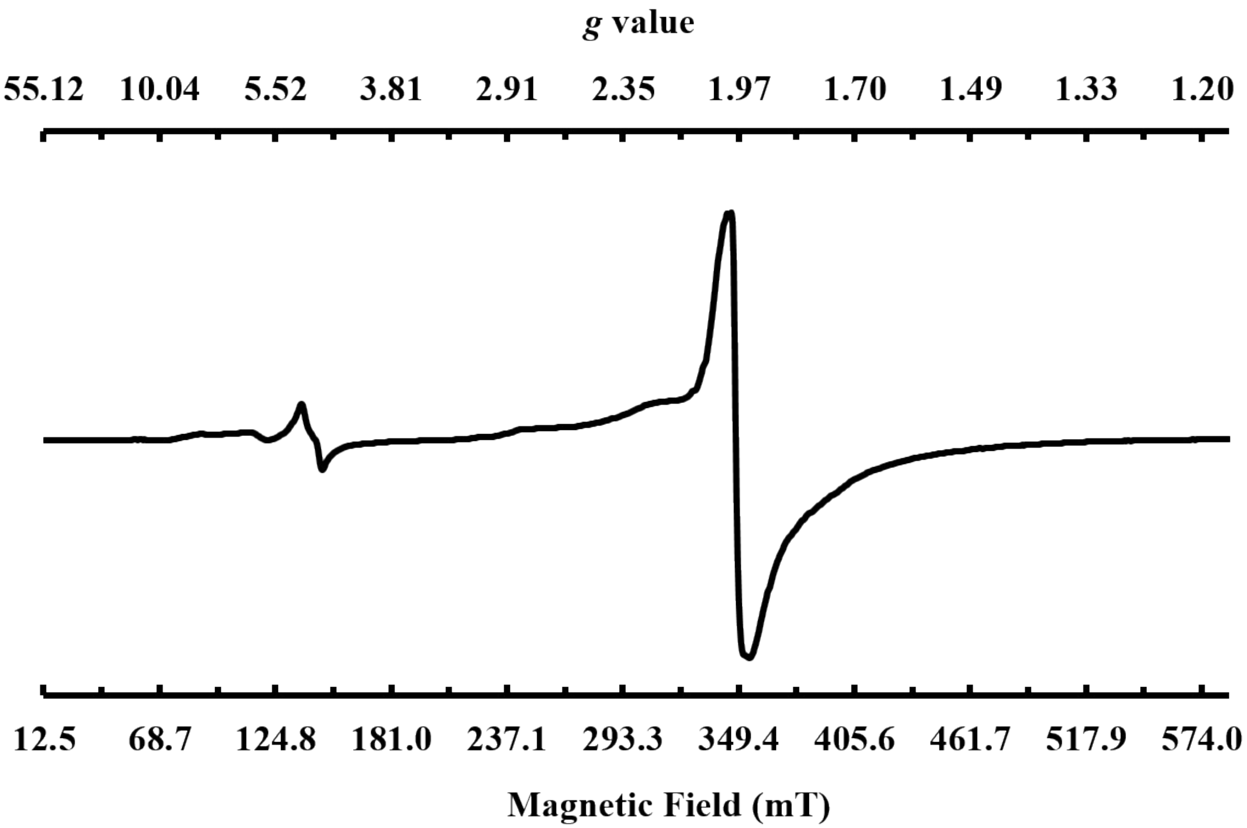

Figure S14. Perpendicular mode X-band EPR spectra of single crystals of **2-Gd/3-**Gd dissolved in THF (1 mM) at 10 K ($g_1 = 4.788$, $g_2 = 1.985$; ν = 9.644 GHz; P = 0.2026 mW; modulation amplitude = 10.02 mT).

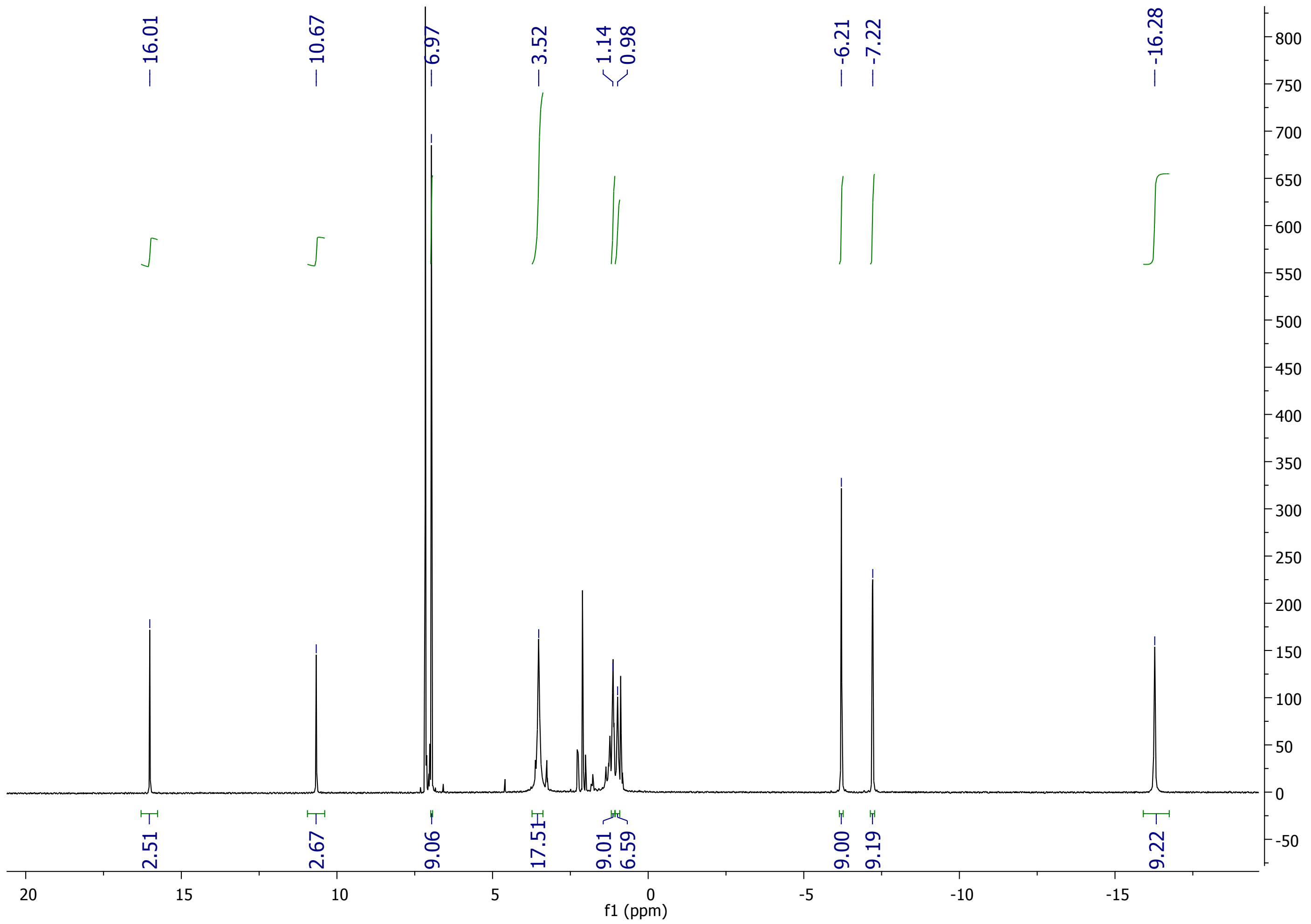

Figure S15. ^{1}H NMR spectrum of **1-Nd** in C_6D_6 (~15 mM) at 298 K.

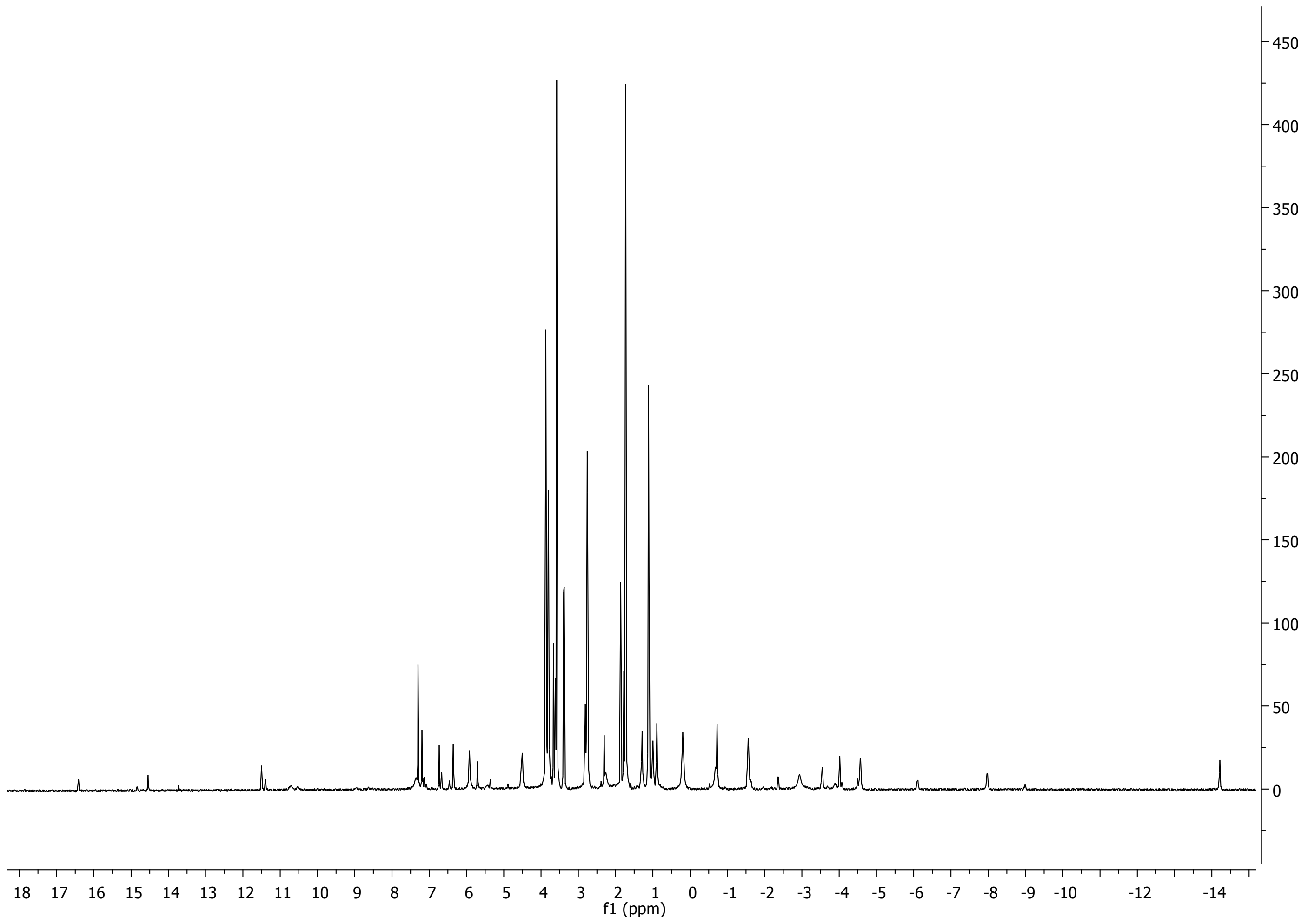

Figure S16. ^{1}H NMR spectrum of **2-Nd** in THF-d_8 (~5 mM) at 298 K.

Computational Details

The initial geometries for complexes **1-Ln** and **2-Ln**, obtained from their respective crystal structures, were optimized using the Tao-Perdew-Staroverov-Scuseria (TPSS) functional.[13] Dispersion effects were incorporated using the semi-empirical D3 dispersion corrections.[14] In the case of **2-Ln**, the optimizations were carried out after removing the countercations. Small-core quasi-relativistic effective potentials[17] were used to model the innermost 28 core electrons of the Ln atoms. The solvation effects were modeled using the COSMO continuum solvation model.[18] A dielectric constant of 7.52, corresponding to tetrahydrofuran solvent, was used. All calculations were carried out in C1 symmetry using at least m3 fine grids. The ground state energies were converged to 10^{-6} a.u and the gradients were converged to 10^{-3} a.u. Vibrational analyses were performed to confirm that all structures were minima on the molecular potential energy surface. All calculations were performed using the Turbomole quantum chemistry software.[19]

For **2-Nd**, the geometries were optimized using def2-TZVP[15] basis sets for the Ln and O atoms, and def2-SVP[16] basis sets for the C and H atoms. The quintet spin-state was established to be the ground state; the doublet configuration was found to be 13.4 kcal higher in energy than the quintet. The geometrical parameters of the optimized quintet structure were in good agreement with the experiments and matched better than the doublet further conforming that the ground state for **2-Nd** is a quintet. Vibrational analysis revealed three low-frequency vibrational modes for **2-Nd** to be imaginary but correspond to rotations of methyl groups located on the adamantyl groups.

In the case of **2-Gd,** we noticed that the spin-state ordering was sensitive to the choice of basis sets. We therefore carried out basis-set convergence studies. At the largest basis-set considered, we found that the nonet spin-state has the most stable electronic configuration which is in agreement with the EPR data. We used def2-QZVP basis on the Gd atom, def2-TZVP on the O atoms and the mesitylene carbon atoms, and def2-SV(P) basis on all other atoms. The septet state is 16.7 kcal/mol higher in energy at the present level of theory. The potential energy as a function of the Gd-centroid distance appears to have two minima. For both spin states, the minimum with longer Gd-centroid distance is preferred according to the calculations, and corresponds to a Gd^{2+} configuration, where the extra electron is in an orbital with mostly 6s and some 5d character. The LUMO is a mesitylene pi* orbital. For both spin states, the minimum with shorter Gd-centroid distance resembles an ionic configuration with a formal Gd^{3+} ion interacting with a negatively charged mesitylene ligand. The computed S^2 value for the nonet was 20.034, indicating negligible spin contamination. Vibrational analysis confirmed that the structure corresponds to a minimum.

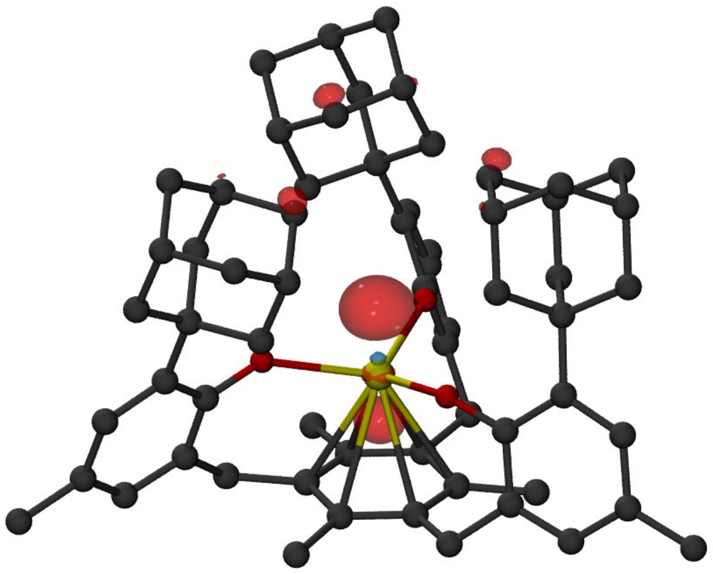

Figure S17. Isosurface for the lowest d-type unoccupied orbital **2-Nd** with a contour value of 0.05. Hydrogen atoms are omitted for clarity.

Table S5. Comparison of selected bond lengths (Å) and angles (°) of **2-Nd**, **1-Nd**, and **2-Gd** obtained from experimental crystal structures with those from DFT calculations in solution phase.

**Compound**	**2-Nd**			**2-Gd**			**1-Nd**	
	Expt.	Calc. (S=2)	Calc. (S=1)	Expt.	Calc. (S=4)	Calc. (S=3)	Expt.	Calc. (S=3/2)
**M–O**	2.237(4)	2.28	2.24*	2.203(3)	2.24	2.21*	2.19	2.19
**M–Cent**	2.366	2.37	2.32	2.286	3.11	2.99	2.489	2.48
**M–C_{arene}**	2.742(6),<br>2.788(7)	2.74<br>2.79	2.75<br>2.78<br>2.70<br>2.79<br>2.74<br>2.74	2.672(5)<br>2.710(6)	3.43<br>3.39	3.32<br>3.30<br>3.30<br>3.27<br>3.35<br>3.30		2.82<br>2.85<br>2.83<br>2.88<br>2.92<br>2.86
**M–C_{arene} avg**	2.765	2.77	2.75	2.691	3.41	3.30		2.86
**M_{oop}**[a]	0.530	0.49	0.54	0.578	-0.28	0.61	0.268	0.24
**∡O-M-O**	114.59(8)	115.6	115.3*	113.37(8)	118.4*	119.5*		118.9*
**Largest C_6 Torsion Angle (°)**	6.2	6.9	12.3	8.8	5.7	6.18	5.6	6.1

*Indicates averaged values.

Table S6. Mulliken population analysis of singly occupied orbitals of **2-Nd** and **2-Gd**.

		Total	s	p	d	f	g
				**2-Nd**			
SOMO-3	Nd	0.99130	0.01090	0.00089	-0.00003	0.97952	0.00001
SOMO-2	Nd	0.95718	0.01239	-0.00009	0.00671	0.93817	0.00001
SOMO-1	Nd	0.78501	0.00000	-0.00032	0.02173	0.76349	0.00011
	C^{mes}	0.18178	0.00306	0.17707	0.00164		
SOMO	Nd	0.78297	0.00000	-0.00032	0.02188	0.7613	0.00011
	C^{mes}	0.1875	0.00302	0.18029	0.00417		
				**2-Gd**			
SOMO	Gd	0.83585	0.62813	0.01506	0.18062	0.01201	0.00003

Table S7. Mulliken atomic spin density analysis of **2-Nd** and **2-Gd**.

	Total	s	p	d	f	g
				**2-Nd**		
Nd	3.63878	0.03172	0.01492	0.10303	3.48868	0.00042
O	-0.05041	-0.00744	-0.0444	0.00137	0.00006	
C^{mes}	0.39735	0.04741	0.34335	0.00661		
				**2-Gd**		
Gd	8.05374	0.7377	0.05963	0.3037	6.95281	-0.00009

References

1. APEX2 Version 2014.11-0, Bruker AXS, Inc.; Madison, WI 2014.
2. SAINT Version 8.34a, Bruker AXS, Inc.; Madison, WI 2013.
3. Sheldrick, G. M. SADABS, Version 2014/5, Bruker AXS, Inc.; Madison, WI 2014.
4. Sheldrick, G. M. SHELXTL, Version 2014/7, Bruker AXS, Inc.; Madison, WI 2014.
5. International Tables for X-Ray Crystallography 1992, Vol. C., Dordrecht: Kluwer Academic Publishers.
6. (a) Spek, A.L. SQUEEZE, Acta Cryst. 2015, C71, 9-19., (b) Spek, A. L. PLATON, Acta. Cryst. 2009, D65, 148-155
7. APEX2 Version 2014.1-1, Bruker AXS, Inc.; Madison, WI 2014.
8. Sheldrick, G. M. SADABS, Version 2014/4, Bruker AXS, Inc.; Madison, WI 2014.
9. Sheldrick, G. M. SHELXTL, Version 2014/6, Bruker AXS, Inc.; Madison, WI 2014.
10. Parsons, S.; Flack, H. D.; Wagner, T. Acta. Cryst., B69, 249-259, 2013.
11. APEX2 Version 2014.9-0, Bruker AXS, Inc.; Madison, WI 2014.
12. Neese, F. Electronic Structure and Spectroscopy of Novel Copper Chromophores in Biology, Diploma thesis, Universität Konstanz, 1993.
13. J. Tao, J. P. Perdew, V. N. Staroverov, and G. E. Scuseria, *Phys. Rev. Lett.*, 2003, **91**, 146401.
14. S. Grimme, J. Antony, S. Ehrlich, and S. Krieg, *J. Chem. Phys.*, 2010, **132**, 154104.
15. F. Weigend and R. Ahlrichs, *Phys. Chem. Chem. Phys.*, 2005, **7**, 3297-3305.
16. A. Schafer, H. Horn and R. Ahlrichs, *J. Chem. Phys*., 1992, **97**, 2571-2577.
17. D. Andrae, U. Hauessermann, M. Dolg, H. Stoll and H. Preuss, *Theor. Chim. Acta*., 1990, **77**, 123-141.
18. A. Klamt and G. J. Schueuermann, *Chem. Soc., Perkin Trans. 2*, 1993, 799.
19. F. Furche, R. Ahlrichs, C. Hattig, W. Klopper, M. Sierka and F. Weigend, WIREs Comput. Mol. Sci., 2014, **4**, 91-100.
